# The impact of the Brazilian family health on selected primary care sensitive conditions: A systematic review

**DOI:** 10.1371/journal.pone.0182336

**Published:** 2017-08-07

**Authors:** Mayara Lisboa Bastos, Dick Menzies, Thomas Hone, Kianoush Dehghani, Anete Trajman

**Affiliations:** 1 Graduate Internal Medicine Program, Federal University of Rio de Janeiro. Rio de Janeiro (RJ), Brazil; 2 Respiratory Epidemiology & Clinical Research Unit, McGill University. Montreal (QC), Canada; 3 Department of Primary Care and Public Health, School of Public Health, Imperial College. London, United Kingdom; 4 Social Medicine Institute, Rio de Janeiro State University. Rio de Janeiro (RJ), Brazil; Leibniz Institute for Prvention Research and Epidemiology BIPS, GERMANY

## Abstract

**Background:**

Brazil has the largest public health-system in the world, with 120 million people covered by its free primary care services. The Family Health Strategy (FHS) is the main primary care model, but there is no consensus on its impact on health outcomes. We systematically reviewed published evidence regarding the impact of the Brazilian FHS on selective primary care sensitive conditions (PCSC).

**Methods:**

We searched Medline, Web of Science and Lilacs in May 2016 using key words in Portuguese and English, without language restriction. We included studies if intervention was the FHS; comparison was either different levels of FHS coverage or other primary health care service models; outcomes were the selected PCSC; and results were adjusted for relevant sanitary and socioeconomic variables, including the national conditional cash transfer program (*Bolsa Familia*). Due to differences in methods and outcomes reported, pooling of results was not possible.

**Results:**

Of 1831 records found, 31 met our inclusion criteria. Of these, 25 were ecological studies. Twenty-one employed longitudinal quasi-experimental methods, 27 compared different levels the FHS coverage, whilst four compared the FHS versus other models of primary care. Fourteen studies found an association between higher FHS coverage and lower post-neonatal and child mortality. When the effect of *Bolsa Familia* was accounted for, the effect of the FHS on child mortality was greater. In 13 studies about hospitalizations due to PCSC, no clear pattern of association was found. In four studies, there was no effect on child and elderly vaccination or low-birth weight. No included studies addressed breast-feeding, dengue, HIV/AIDS and other neglected infectious diseases.

**Conclusions:**

Among these ecological studies with limited quality evidence, increasing coverage by the FHS was consistently associated with improvements in child mortality. Scarce evidence on other health outcomes, hospitalization and synergies with cash transfer was found.

## Introduction

Primary healthcare is essential for progress towards Universal Health Coverage [1], a key Sustainable Development Goal [2], which includes the provision of promotion, prevention, treatment, rehabilitation and palliative care to all citizens without significant financial burden [3]. Primary healthcare can contribute to Universal Health Coverage through expanding access to cost-effective, population-based healthcare and addressing the wider social determinants of health [4]. Further understanding of the impact of primary healthcare is essential to convincing policy-makers to invest in and expand it.

Brazil is an excellent setting for evaluating the impact of primary healthcare and Universal Health Coverage. Following health reform in the 80’s, Brazil has been expanding access to healthcare through the Unified Health System (SUS—*Sistema Único de Saúde*). Expansion of the SUS has been in line with the key principles outlined in the 1988 Brazilian Constitution of universalization, equity, and comprehensive care [5]–also core foundations of Universal Health Coverage [6]. The SUS can be considered the largest, universal, free-of-charge public health system in the world. Nearly 160 million people (76% of the Brazilian population) [7] depend exclusively on SUS services. Despite many limitations, the SUS is a major step towards Universal Health Coverage in Brazil [8].

At the heart of the SUS, is the Family Health Strategy (FHS) (*Estratégia Saúde da Família* in Portuguese), which is the main primary healthcare model in the country. It emphasizes health care in community health facilities and at home to a defined local population. Services are provided by family health teams composed of one physician, one nurse, one nurse aide, and from four to twelve full-time community health workers. Each team is responsible for up to 1000 families, or 3500–4500 people [9]. The FHS is progressively replacing the traditional primary healthcare units, which are based on physician-centered care, by either general practitioners or specialists.

The FHS has grown from previous healthcare models including the Community Health Agent Program (PACS, from *Programa de Agentes Comunitários de Saúde* in Portuguese) that was established by the Ministry of Health in 1994, which then became the Family Health Program in the late 1990s. In 2011, the Family Health Program was renamed FHS to place primary healthcare at the center of the SUS [10]. The population covered by the FHS has progressively increased from 7% in 1998 to 63% in 2015, corresponding to almost 120 million people [11, 12]. The FHS has expanded in a stepwise way across municipalities and across the country providing an opportunity for evaluation utilizing reasonable quality national databanks [13–16]. During expansion, priority catchment areas included preferentially the most vulnerable populations (poor neighborhoods, including slums) [12]. Concurrent with FHS expansion, conditional cash transfer programs were gradually implemented in the country as poverty-alleviating programs. In 2003, these programs were consolidated into the *Bolsa Família* Program, which includes specific health conditionality such as prenatal care, child development follow up and immunization [17]. The impact on health outcomes of these two large and interdependent national health programs is poorly understood.

There is evidence that primary healthcare in general can improve health outcomes–particularly child health, and infectious and cardiovascular diseases [18, 19]. However, although many studies of the impact of FHS on different outcomes have been published, results are inconsistent and have not been systematically reviewed previously. We undertook a systematic review to answer the following questions: (1) What is the impact of the Brazilian FHS expansion on primary health care health outcomes? (2) Is the FHS superior to other models of primary health care with regard to these outcomes? And (3) Does the *Bolsa Familia* Program enhance the effect of the FHS Program?

## Material & methods

### Literature search strategy

We searched three electronic databases [Medline (through PubMed), Web of Science and Lilacs] for records that included “Brazil” and “Family Health Strategy” (or multiple synonyms of these) and any relevant health outcomes in title or abstract, as key words, or as Medical Subject Heading (MeSH) term (see support information for PRISMA checklist and S1 Table with full search strategy). Relevant health outcomes included tuberculosis, child nutrition, infant mortality, low birth weight, infant diarrhea, breastfeeding, vaccination, AIDS/HIV, sexually transmitted diseases, dengue, neglected tropical diseases, prenatal care, and hospitalizations. Selected health outcomes were based on known primary healthcare sensitive-conditions [20]. Hospitalization due to conditions sensitive to primary care was defined according to the Brazilian Ministry of Health list [21], based on the 10^th^ International Disease Classification. In Lilacs, the search strategy was conducted in Portuguese, MeSH terms in Portuguese (“Descritores em Saúde”– www.bireme.br) and their synonyms were used. The search was limited to the period from January 1994 (when the FHS started) until May 2016. No language restriction was used in the search. Lilacs includes theses from Brazilian Universities, and these were included.

### Study selection

Titles, abstracts and full texts were screened by two independent reviewers (MB and AT), with consensus in each stage. A third reviewer (DM) was consulted to resolve disagreements.

Records were included if: (i) the population was Brazilian; (ii) the intervention evaluated was the FHS; (iii) there was a comparator of any form including different levels of population coverage of FHS (over time or geographical) or other primary health care models; (iv) at least one of the health outcomes cited above was measured and reported as one of the following absolute rates: incidence, prevalence or mortality rates and hospitalization. Treatment outcomes and vaccination coverage were also included. Studies that reported only process indicators (such as number of pre-natal visits or directly-observed treatment for tuberculosis) were not included; (v) the final results were adjusted for at least one of the following group of relevant variables (potential confounders of health outcomes): socioeconomic (e.g. education or socioeconomic status), sanitary condition (e.g. water supply and sewage) or health system access (e.g. number of physicians, or number of hospital beds); and vi) the study design was either experimental or observational. Reviews, opinions, and case reports were not included.

### Statistical analysis and data synthesis

Since there was overlap of study populations (same set of patients, in the same range of time and area) and heterogeneity in methods employed pooling of data was not attempted. Descriptive results for each study are reported in the supplemental material. We summarized the main results of each study by groups of outcomes. Whenever there was sufficient detail in the manuscript, we converted effect sizes into rate ratio outcomes in order to make outcome measures comparable. When infant mortality was divided into two age periods [neonatal (i.e. death of infant aged up to 27 days and post neonatal (i.e. death of infant aged 28 days to 1 year)], we presented stratified data.

### Assessment of methodological quality

Two reviewers (MB and TH) assessed the quality of each included study using a published checklist for observational and longitudinal studies [22]. This checklist was chosen as all studies were observational in nature and a large proportion (21 of 31 studies) utilized longitudinal methods. Disagreements in scoring between reviewers were resolved by discussion. Since the majority of studies used an ecological design, with geographical areas as the unit of analysis, we excluded three questions related to consent of participants. The remaining 30 questions were scored with two points for “yes” (indicating a lower risk of bias), one point if the answer was “unclear” or zero if the answer was “no” (higher risk). The final scores were obtained by summing questions, studies grouped into “generally higher quality” or “generally lower quality” based on the distributions of the scores obtained in this review. Studies scoring under 37 (out of 60) were considered low-quality.

## Results

### Study selection and major methodological issues

**Fig 1:**
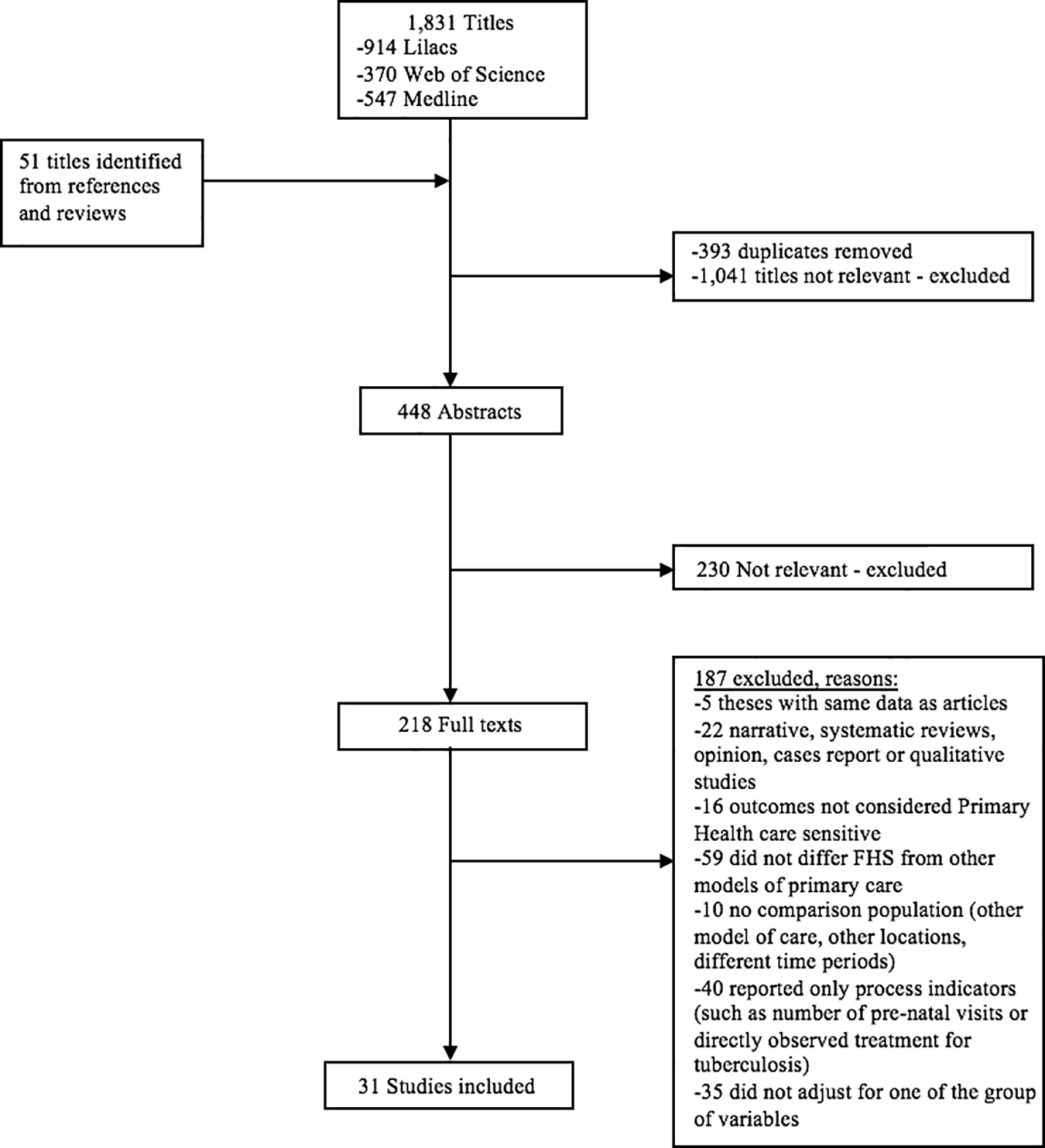
Flow chart of study selection.

Of 1,831 publication titles found in the search, 218 studies were selected for full text review, from which 31 studies were selected that met our inclusion criteria (Fig 1).

There was considerable variability concerning the units of analysis, the geographical regions included, methods of measurement of FHS coverage, and the outcome measured. Among the 31included studies [23–53], six used individual-level data [34, 47–49, 51, 52], with samples ranging from 961 to 7,534 subjects, whilst the remaining 25 studies were ecological analyses. Most (n = 19) ecological studies used municipalities as the unit of analysis, but three chose to analyze micro-regions (set of several neighbor municipalities) [25, 32, 44] or states [31, 37] (S2 Table). For these studies, potential ecological biases can be greater due to large differences in socio-economic characteristics between micro-regions, macro-regions, and states (e.g., the North and Northeast regions are poorer and less developed than the Southern and Southeast regions) [54].

The method of estimation of the proportion of the population covered by the FHS was different in the ecological studies. In six of them [27, 33, 37, 40, 46, 53], it was estimated by multiplying the number of family health teams by 3500 (estimated population covered by one team) while seven considered persons actually registered by the family health clinic [24, 30, 35, 38, 39, 41, 50]. The method was not specified in the 12 remaining ecological studies. No ecological study estimated the proportion of persons actually using the FHS clinics. Among the six individual-level studies, three studies [48, 49, 51] compared regular users of the service versus users of other models of primary health care.

In addition to differences in definitions of coverage, the classification of FHS coverage varied considerably including categorical ranges (e.g. <30%, 30–69%, >70%), as a continuous variable (i.e. percentage of the population covered), or simply availability or not of FHS in the municipality (S2 Table). Only four studies compared FHS with the traditional model of primary care [47–49, 51].

The outcome measurements and reported results were also highly variable (e.g. the proportion of reduction in mortality rate, number of deaths reduced per 1000 live births, and change in slope of association).

Of 21 studies that started after at least two years of the *Bolsa Família* Program implementation, only four [26, 30, 35, 50] examined the effect of this cash transfer program on health outcomes.

### Quality of studies

All studies were observational in nature, with low overall quality of evidence. The lowest scoring study was 31, and the highest 45 (out of 60). Areas of potential bias that a high proportion of studies failed to address were justification of sample size (a risk of bias in conducting under-powered studies), reliability of outcomes and measurements used (a risk of measurement bias), issues of missing data and observations per time point, reporting of absolute effect sizes (most reported only relative effect sizes), quantitative assessment of any bias, and issues of generalizability (S1 Fig).

Of the 31 studies, we classified 10 into generally lower quality, and 21 as higher qualities. There was a general clustering of scores around 40–43 (S3–S5 Tables).

### Impact on child mortality (n = 14, Table 1 & S3 Table) [23–36]

Fourteen included studies examined the association between FHS coverage and mortality of children, of which five analyzed the effect on both neonatal (0–28 days) and post-neonatal (28 days to one year) mortality [23, 25–28], two only post-neonatal mortality [29, 30], one both infant mortality (0–1 year) and child (under 5) mortality [33], three just infant mortality [31, 32, 34], two just child mortality [35, 36], and one study reported neonatal, post-neonatal and child mortality [24]. All of these studies, apart from one [26], were scored of higher quality (S3 Table).

**Table 1:** Summary of results of included studies reporting neonatal (up to 27 days), post-neonatal (28 days- 1 year), infant (neonatal + post-neonatal) and child (up to 5 years) mortality (n = 14*).

Outcome	N	Study Population	Effect in the Outcomes			Range of Effect Observed**
			Improved	No effect	Worse	
Neonatal Mortality	6	– 5 studies: municipalities (range: 110–5,506 cities)	4^23,24,27,28^	2^25,26^	-	Ecological
						– 2 studies: 11%-19% decrease in areas with ≥70% of FHS coverage
		– 1 study: all Brazilian micro-regions (n = 558)†				– 1 study: 7.8%-13.8% decrease comparing areas with FHS vs. no FHS over a 3-year period
						– 1 study: decrease of 0.8/1,000 live births for each 10% increase of coverage.
Post-Neonatal Mortality	8	– 7 studies: municipalities (range: 110–5,506 cities)	7^23−26,28–30^	1^27^	-	Ecological
						– 3 studies: 17%-66% decrease in areas with ≥70% of FHS coverage.
		– 1 study: all Brazilian micro-regions (n = 558)†				– 3 studies: 0.8%-6% decrease for each 10% of increase of FHS coverage.
						– 1 study: 1.2%-9.8% decrease in areas with FHS vs. no FHS over a 3-year period.
Infant Mortality	4	– 1 study: all 26 Brazilian states	3^31−33^	1^34^	-	Ecological
						– 2 studies: ~5% decrease for each 10% increase of FHS coverage.
		– 1 study: 35 micro-regions from one state†				– 1 study: 3% decrease for each additional year after the municipality adopted the FHS.
		– 1 study: 4,488 municipalities				Individual level
						– No effect.
		– 1 study: Individual level (2,144 children)				
Child Mortality	4	– 4 studies: municipalities (range: 224–4,488 cities)	4^24,33,35,36^	-	-	Ecological
						– 2 studies: ~13% decrease in areas with ≥70% of FHS coverage
						– 1 study: 5% decrease for each 10% increase in FHS
						– 1 study: 3% decrease or each additional year in the FHS

Notes

*5 studies reported both neonatal and post-neonatal mortality; 1 study reported neonatal, post-neonatal mortality and child mortality, 1 study reported both infant mortality and child mortality.

†Micro-regions: Set of several neighbor municipalities- (according with the Brazilian 2010 census the average population for micro-region is ~342,000)

** If no change–then results not summarized

**Abbreviations:** FHS: Family Health Strategy

There was an association between higher FHS coverage and lower mortality in most studies (13 out of 14) across different age stratifications within child mortality. No studies reported increased mortality from FHS coverage. Of the six studies examining neonatal mortality, lower mortality was associated with increased FHS coverage in four. Of the eight studies examining the post-neonatal period, only one showed a non-significant finding, with the reductions in mortality rates ranging from 0.8% to 66%, depending on the level of coverage. Of four studies examining infant mortality, only one found a non-significant effect of FHS (Table 1).

Only one study employed individual-level data and reported no association between infant mortality and FHS coverage [34] (Table 1).

Three studies analyzed the period after the *Bolsa Família* Program implementation. All three [26, 30, 35] studies showed that FHS had an independent beneficial effect beyond improvements in health from the *Bolsa Família* Program. Two [26, 30] studies reported that the *Bolsa Família* Program effects were synergistic with the FHS (i.e. there were greater reductions in mortality from FHS coverage with higher *Bolsa Família* coverage). No studies that evaluated infant mortality compared the FHS with other models of primary care.

### Impact on hospitalization for primary care sensitive causes (n = 13, Table 2 & S4 Table) [34, 36–47]

There was variability in the selected conditions, categorization of exposure, target population, and outcomes measured in the 13 studies analyzing hospitalizations for primary care sensitive conditions. Four [41, 43, 46, 47] of these studies were considered of generally lower quality (S4 Table).

**Table 2:** Summary of results of included studies reporting hospitalization due to primary care sensitive causes* (n = 13).

Outcome	N	Study Population	Effect in the Outcomes			Range of Effect Observed**
			Decrease	No effect	Increase	
Hospitalizations due to primary care sensitive causes (diseases not specified)	6	– 2 studies: municipalities (range 78–188 cities)	3^37,40,44^	2^41,47^	1^45^	Ecological
						– Decreased -3 studies: 4%-10% decrease in areas with FHS≥70% coverage
		– 1 study: All states of Brazil (26 States)				– Increased—1 study: increase by 1.0/10,000 habitants for additional year in the FHS
		– 1 study: All Brazilian micro-region (n = 558)†				Individual level
						– No effect
		– 1 study: 1,909 census tract in one municipality				
		– 1 study: Individual level (1,058 patients)				
Hospitalizations due to chronic diseases	4	– All studies: municipalities (range: 137–5,507 cities)	4^38,39,42,43^	-	-	Ecological
						– Decreased
(e.g. diabetes, cardiovascular)						2 studies: 13%-30% decrease in areas with high coverage of FHS
						2 studies: 0.71–11% decrease for each 10% increase of FHS coverage, but only in women for selected outcomes (diabetes and myocardium infarction)
Hospitalizations due to diarrhea and lower respiratory infection	3	– 2 studies: municipalities (range: 12–224 cities)	1^34^	2^36,46^	-	Ecological
						– No effect
		– 1 study: Individual level (2,144 children)				Individual level
						– Decreased -1 study: 70% decrease when children were covered by FHS (due diarrhea) ††

Notes

* Primary care sensitive causes included 20 groups of diagnosis available *Alfradique et al*^*21*^

†Micro-regions: Set of several neighbor municipalities (according with the Brazilian 2010 census the average population for micro-region is ~342,000)

** If no change–then results not summarized

†† Had no effect in the rates of hospitalization for respiratory infection.

**Abbreviations:** FHS: Family health strategy

Of these studies, eight found a decrease in the hospitalization rates associated with increased FHS coverage, although in two [42, 43] the effect was seen only in women for diabetes and cardiovascular diseases. The reported effect sizes ranged from 0.71–11% for each 10% increase in FHS coverage. Only one [45] study showed increased hospitalizations from FHS expansion (Table 2). Of the four studies that showed no significant effects [36, 41, 46, 47], three [41, 46, 47] were considered of generally lower quality. All studies that showed decreased or increased in hospitalizations rate, apart from one [43], were scored as high quality.

Two [34, 47] studies had individual level design, one [34] of which associated a decrease in hospitalizations of children due to diarrhea with higher FHS coverage (Table 2).

Eleven studies analyzed the period after the *Bolsa Família* Program implementation, but none of these studies included the *Bolsa Família* as a co-variable in their regression models. Only one study [47] compared the FHS with other primary care models and no difference was observed (S4 Table).

### Impact on other health outcomes (n = 7, Table 3 & S5 Table) [34, 48–53]

Seven studies examined other health outcomes beyond child mortality and hospitalizations for primary health care sensitive conditions. All but two studies [34, 50] were considered of lower quality (S5 Table).

**Table 3:** Summary of results of included studies for all other health outcomes (n = 7*).

Outcome	N	Study Population	Effect in the Outcomes			Range of Effect Observed**
			Improved	No Effect	Worse	
Prenatal care	2	– 2 studies individual level: 961–2,144 population size	1^48^	1^34^		Individual Level
						– 1 study: 44% increase in vaccination coverage in pregnant women who attended prenatal care in FHS. 100% increase in maternal admission when prenatal care was in FHS. No effect on proportion with low birth weigh
Vaccination coverage	2	– 2 studies individual level: range 2,144–7,534 interviews	-	2^34,49^	-	-
Infectious Diseases (Leprosy, Congenital Syphilis and Tuberculosis)	3	– 2 studies: municipality (range 897–1,358 cities)	1^50^	1^51^	1^53^	Ecological Level
						– 1 study: 12% increase in the detection rate of Leprosy when FHS>70%
		– 1 study: Individual level:1,396 patients				– 1 study: 0.04/10,000 more cases of congenital syphilis for each 10% increase of FHS coverage
						Individual Level
						– No Effect
Child Malnutrition	1	– 1 study: Individual level: 3,931 children	1^52^	-	-	Individual Level
						– 1 Study: Odds of child malnutrition were 48% lower in areas with FHS coverage of 50%.

Notes

* One study reported both prenatal care (low weight birth outcome), and vaccination coverage

** If no change–then results not summarized

**Abbreviations:** FHS: Family health strategy.

Two studies [34, 48] examined a range of pre-natal care outcomes. Only in one [48] study, which was of lower quality, and for only one outcome (tetanus vaccination) was the FHS coverage associated with better outcomes in prenatal care: a 44% increase in the vaccination coverage in pregnant women, when compared with another model of primary care. No effect was observed in the other two studies that reported vaccination coverage. A small increase of 0.04 per 1000 cases (CI 95% 0.003; 0.07) of congenital syphilis was observed for each 10% increase of FHS coverage [53]. There was an association between increased leprosy detection [50] and lower child malnutrition [52] with increasing FHS coverage. No effect in tuberculosis end of treatment outcomes was observed when FHS was compared with other models of primary care [51] (Table 3).

Five studies [34, 48, 49, 51, 52] had an individual-level design; in two of them the FHS had a positive effect: on child malnutrition [52] and on tetanus vaccination [48] (Table 3).

Only one [50] study evaluated the *Bolsa Família* Program together with FHS, and reported a greater detection of leprosy cases when coverage by both the *Bolsa Família* Program and the FHS were higher (S5 Table).

Finally, none of the studies that met our inclusion criteria reported on breast-feeding, dengue, HIV/AIDS or other neglected infectious diseases.

## Discussion

There is reasonable evidence that increased coverage of FHS is associated with lower infant (mainly post-neonatal) and child mortality. The association between FHS coverage and hospitalizations from primary care sensitive conditions was less clear, but many studies reported reductions in hospitalization rates. Although the FHS was also associated with improvements in other health outcomes such as child malnutrition, congenital syphilis, leprosy detection and prenatal anti-tetanic vaccination, the scarce number of publications and greater risk of bias in these studies precludes generalization.

Infant and child mortality were the most frequently studied outcomes, potentially explaining the generally more reliable evidence and consistent associations. FHS coverage was associated with larger reductions in post-neonatal mortality than in neonatal mortality, which may be expected given neonatal deaths are often related to birth complications or congenital conditions and less affected by improvement of primary care assistance [55, 56]. However, some studies did show some reduction in mortality in the neonatal period, which may be explained with better pre-natal care and timely referral to secondary care. The *Bolsa Família* Program and literacy of adults had a similar independent effect on infant mortality, sometimes stronger than the FHS effect [30, 31, 35]. The greater association between FHS coverage and improved health outcomes when *Bolsa Familia* was present is expected given the compulsory usage of healthcare services (for example childhood check-ups) to receive benefit payments.

The relationship between FHS coverage and hospitalizations for primary care sensitive conditions was less clear, although a majority of studies found hospitalization rates declined. Municipality selection, baseline conditions, number of existing hospital beds and healthcare workers, selected diagnoses and confounder adjustment may explain the differences between findings. Unfortunately, elucidating potential explanatory trends is beyond the ability of this review. Understanding the impact of the FHS on hospitalizations is important given hospitalizations are a major driver of health costs. The theory underpinning the relationship between primary health care and hospitalizations is complex and may partially explain the lack of clarity in the findings. Whilst primary care sensitive hospitalizations should fall with increased access to primary health care, this may not be the case if the services are weak (e.g. the technical capacity of staff is insufficient) or primary care sensitive conditions are in an advanced stage. In this case, hospitalizations may increase. On the other hand, the effect for chronic diseases such as hypertension or diabetes is likely to involve long time lags. Finally, hospitalization rates depend also on bed availability, not examined in the current study.

There has been significant improvement in socioeconomic and sanitary conditions in Brazil in the last three decades, which are likely to explain many improvements in health. To address this, we only included studies that adjusted for relevant socioeconomic, sanitary or health access conditions, which excluded 35 studies.

Regardless of the effect, only a minority of studies compared FHS with other models of primary care, so we cannot make conclusions on benefits from the FHS model or coverage compared to other models of primary care. The four studies that compared FHS versus other models [47–49, 51] showed no differences in the majority of outcomes. Even so, there is large variation in historical primary health care services across Brazil and between urban and rural areas. Many are fragmented, under philanthropic or private control, exist as part of hospital systems, or do not exist at all. Moreover, parts of both the FHS covered and non-covered population have access to private healthcare which constitute 24% of the Brazilian population [7]. This is likely to make the relationship between FHS coverage and health outcomes more complex and was insufficiently addressed in the studies. The lack of clarity in actual control or comparator services also clouds the ability to draw comparisons, and raises the question “what are we exactly comparing the FHS to?” The FHS is a highly standardized program with mandated components from the Federal level, so in some areas the FHS program is an expansion of services to an unserved population, whilst in other areas, it offers a change in the structure and strategy of primary health care services focusing on outreach, prevention, promotion, and person-centered care. More research is required on which dimensions of the FHS are driving the reported improvements in health.

Our review has a number of limitations. Firstly, our search may have missed important studies. We only searched three electronic databases, and non-peer reviewed articles and reports may have been missed. Secondly, we restricted our searches to a few outcomes, based on the assumption that those were the most primary care sensitive. Thirdly, our assessment of quality and bias was focused towards ecological, longitudinal studies, and only provides a weak understanding of the actual biases present. Inference on the quality of these studies beyond this review is limited. Our findings and analyses were based on weak, ecological studies with the potential for the ecological fallacy, with different methods to estimate the exposure to the FHS. However, many studies used municipalities, with small population sizes, potentially reducing this fallacy. Additionally, many studies employed panel or longitudinal regression, which can be considered of generally better evidence than cross-sectional studies, and can control for temporal changes beyond the FHS. Lastly, the expansion of the FHS was not completely random, with municipal governments opting to provide the FHS services and many clinics built preferentially in poorer neighborhoods [6, 18]. This may confound and bias many studies as unobserved factors determining the FHS uptake may also explain changes in health outcomes. As much of the ecological studies reviewed may suffer from these biases, we cannot consider the evidence conclusive, and studies with more robust methods must be conducted.

Despite these limitations, this is the first critical appraisal, to the authors’ knowledge, of the literature on the effect of the FHS on the health outcomes, and one of only a few systematic reviews examining a national primary care program in a low- or middle-income country. We employed a broad search strategy looking for studies published in different languages, used restriction criteria to ensure only studies of reasonably high quality were included, and considered conditions sensitive to primary care.

Furthermore, the outcomes examined have important implications for other low- or middle-income country’s considering expanding primary health care as part of universal health coverage.

## Conclusion

The FHS expansion was consistently associated with reductions in post-neonatal and child mortality, and less-consistently with reductions in hospitalizations from primary care sensitive conditions. This evidence supports the vital role for primary healthcare in improving health outcomes, and as part of progress towards Universal Health Coverage and the Sustainable Development Goals.

## Supporting information

S1 PRISMA Checklist Checklist for the systematic review. (DOC)

S1 Fig Quality assessment of included studies. (DOCX)

S1 Table PICO question terms used in the search strategy ((Medline, Web of Science, and Lilacs). (DOCX)

S2 Table Summary of methods of included studies. (DOCX)

S3 Table Descriptive results of included studies about neonatal (up to 27 days), post-neonatal (28 days- 1 year), infant (neonatal + post-neonatal) and child (up to 5 years) mortality. (DOCX)

S4 Table Descriptive results of included studies about hospitalization. (DOCX)

S5 Table Descriptive results of included studies for all other health outcomes. (DOCX)
