# Correction: The impact of the Brazilian family health on selected primary care sensitive conditions: A systematic review

**DOI:** 10.1371/journal.pone.0189557

**Published:** 2017-12-07

**Authors:** Mayara Lisboa Bastos, Dick Menzies, Thomas Hone, Kianoush Dehghani, Anete Trajman

The word "strategy" is missing from the article title. The correct title is: The impact of the Brazilian family health strategy on selected primary care sensitive conditions: A systematic review. The correct citation is: Bastos ML, Menzies D, Hone T, Dehghani K, Trajman A (2017) The impact of the Brazilian family health strategy on selected primary care sensitive conditions: A systematic review. PLoS ONE12(8): e0182336. https://doi.org/10.1371/journal.pone.0182336
