# Supplementary material for: The impact of the Brazilian family health on selected primary care sensitive conditions: A systematic review
Source: PLoS One. 2017 Aug 7;12(8):e0182336. doi: 10.1371/journal.pone.0182336 (PMC5546674; doi:10.1371/journal.pone.0182336)
Supplement: S1 Fig — (DOCX) [file pone.0182336.s002.docx]

# Fig S1. Quality assessment of included studies
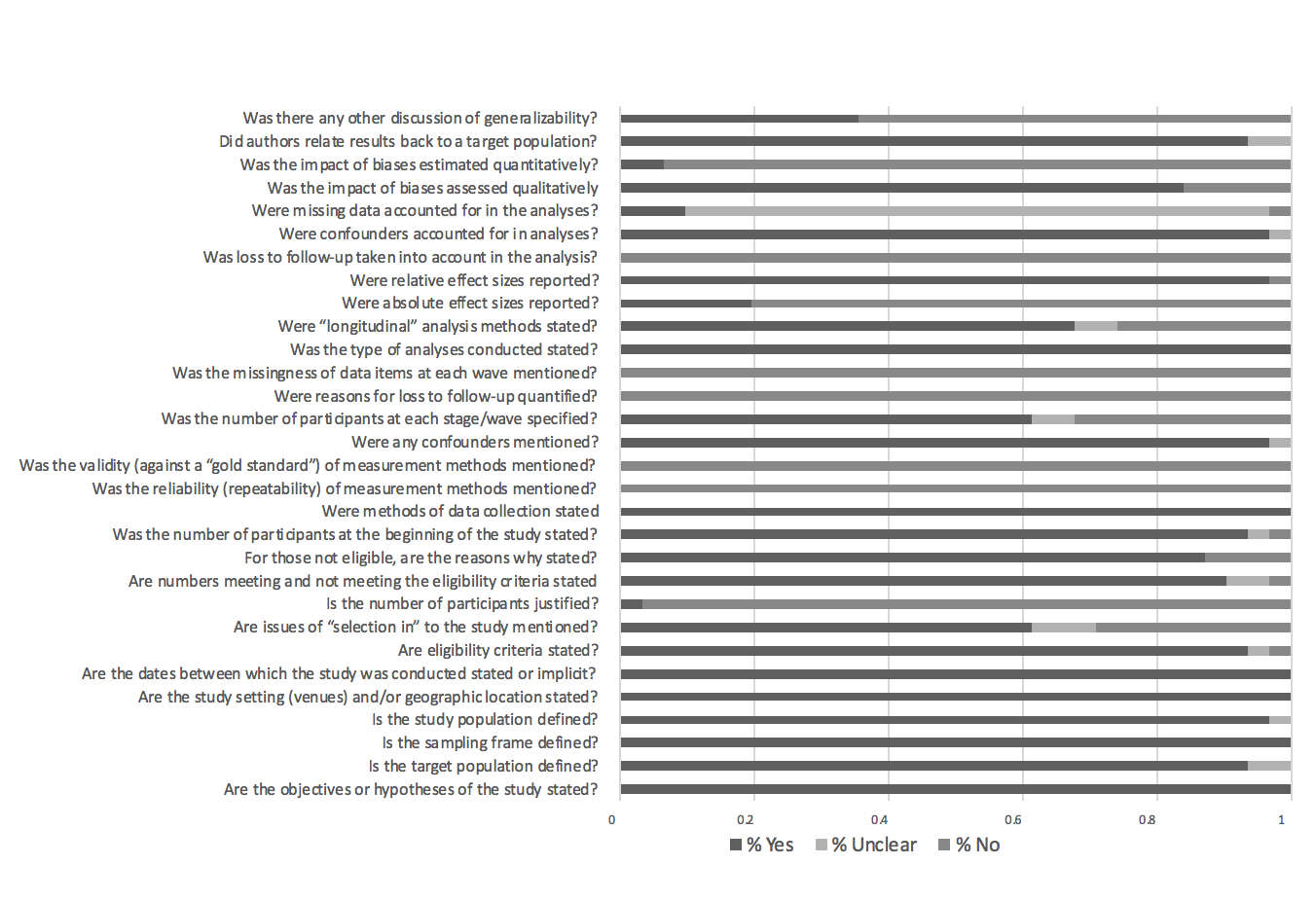


Legend

Reviewers’ assessment for quality and risk of bias. A standardized tool ^15^ for longitudinal observational studies was used. The bars represented the proportion of studies that assessed or not each item of the checklist. The dark part of the bars represents the percentage of studies with low risk of bias, while the light grey part represents the percentage of studies with a high risk of bias.
