# Supplementary material for: The impact of the Brazilian family health on selected primary care sensitive conditions: A systematic review
Source: PLoS One. 2017 Aug 7;12(8):e0182336. doi: 10.1371/journal.pone.0182336 (PMC5546674; doi:10.1371/journal.pone.0182336)
Supplement: S1 Table — (DOCX) [file pone.0182336.s003.docx]

**Supplementary Tables for**

**“**The Impact of the Brazilian Family Health on Selected Primary Care Sensitive Conditions: A Systematic Review**"**

[Table S1 – PICO question and terms used in the search strategy 2](#_Toc488421425)

[Search strategy 4](#_Toc488421426)

[Medline 4](#_Toc488421427)

[Web of Science 5](#_Toc488421428)

[Lilacs 6](#_Toc488421429)

#

| **PICO** | **Mesh** | **Search Terms (English)** | ***Descritores* (Portuguese)** | **Search Terms (Portuguese)** |
| --- | --- | --- | --- | --- |
| **Population** | Brazil | Brazil Brazil* |  | Brazil Or Brazil$ |
| **Intervention** | Family Practice | Family health program  Family health strategy  Primary health care  Family health progra*  Family health strateg*  Family Practice | Medicina de Família e Comunidade | PSF$  ESF$  Saúde da Família  Programa Saúde da Família  Programa de Saúde da Família  Estratégia de Saúde da Família  Estratégia de Saúde na Família  Medicina de Família e Comunidade  Estratégia Saúde da Família  Atenção Básica  Atenção Básica à Saúde  Atenção Primária de Saúde |
|  | Primary Health Care |  | Estratégia de Saúde na Família |  |
|  |  |  | Atenção Primária de Saúde |  |
| **Comparator:**  Other primary model of care or/and FHS coverage over time or geographical |  | | | |
| **Outcome** | | | | |
| Tuberculosis | Tuberculosis | Tuberculosis  LTBI  Latent Tuberculosis  Tuberc*AND (infection OR latent) | Tuberculose | Infecção Tuberculosa Latente  ILTB$  Tuberc$ AND (latent$ OR infec$)  Tuberculose |
|  | Mycobacterium tuberculosis |  | Tuberculose Latente |  |
|  | Latent Tuberculosis |  |  |  |
| Child Malnutrition | Child Nutrition Disorders | Child Nutrition Disorders  Child* AND (Malnutrition OR  Nutrition*) | Transtornos da Nutrição Infantil | Transtornos da Nutrição Infantil  Desnutrição Infantil  Desnutrição em Crianças  Desnutrição da Criança  Desnutri$ AND (Criança$ OR infantil) |
| Infant Mortality | Infant Mortality | (Child* OR Infan*) AND (morta* OR death)  (Morta*OR death) AND (postneonatal OR neonatal) | Mortalidade Infantil | Mortalidade infantil  Mortalidade Pós-neonatal  Mortalidade Neonatal  Transtornos da Nutrição Infantil |
|  | Child Mortality |  | Mortalidade da Criança | Mortalidade da Criança  Mortalidade de Crianças  Mortalidade na Infância  Morta$ AND (neonatal OR infa$ OR Pós-neonatal OR Criança) |
| Low weight birth | Infant, Low Birth Weight | (Low AND Birth AND Weight) AND Infan* | Recém-Nascido de Baixo Peso | Recém-Nascido de Baixo Peso  Baixo Peso ao Nascer  (Baixo AND Peso) AND (Nasc$) |
| Diarrhea Infantile | Diarrhea, Infantile | (Child* OR infan*) AND (Diarrhea OR Gastroenteritis OR Intestinal Parasito*) | Diarreia Infantil  Diarreia  Gastroenterite  Disenteria | (Diarreia OR Gastroenterite OR Disenteria) AND (infan$ OR criança)  Diarreia Infantil |
|  | Diarrhea |  |  |  |
|  | Intestinal Diseases, Parasitic |  |  |  |
|  | Gastroenteritis |  |  |  |
|  | Infant |  |  |  |
| Exclusive Breast Feeding | Breast Feeding | Breast Feed* OR Breastfeed* | Aleitamento materno | Aleitamento materno  (aleitamento AND materno AND exclusivo) |
| Vaccination | Immunization Programs | Vaccine* OR immunization* OR vaccination or vaccina* | Programas de Imunização  Vacinação | Programas de Vacinação  Programas de Imunização  Vacinação  Programa Nacional de Imunizações  Calendário Básico de Vacinação da Criança Calendário de Vacinações  Calendário Nacional de Vacinação Vacina$ |
|  | Vaccination |  |  |  |
|  | Vaccines |  |  |  |
| AIDS and other sexually transmitted diseases | Acquired Immunodeficiency Syndrome  HIV  Sexually Transmitted Diseases | AIDS,  STD,  Sexually Transmitted*,  HIV  (Sexual* AND transmi*)  STDs  Acquired Immunodeficiency Syndrome | Síndrome de Inmunodeficiencia Adquirida  HIV  Doenças Sexualmente Transmissíveis | SIDA  AIDS  Síndrome da Imunodeficiência Adquirida  Síndrome de Imunodeficiência Adquirida  HIV  Doenças Sexualmente Transmitidas  Doenças Sexualmente Transmissíveis  Doenças de Transmissão Sexual  DST$ |
| Neglected Diseases | Neglected Diseases | Neglected Diseases*  Dengue  Lepra  Leprosy  Hansen's disease | Doenças Negligenciadas | Doenças Esquecidas  Doença Negligenciada  Doenças Negligenciadas  Febre da dengue  Dengue  Lepra$ Hanseniase Doença de Hansen |
|  | Leprosy |  | Dengue |  |
|  | Dengue |  | Hanseníase |  |

# Table S1 – PICO question and terms used in the search strategy

| Prenatal | Prenatal Care | Prenatal Care  Prenatal*  Matern* AND (Morta* OR death OR Health OR service) | Cuidado Pré-Natal  Mortalidade Materna | Assistência Pré-Natal  Cuidado Pré-Natal  Pré-Natal$  Mater$ AND Mort$  Mortalidade Materna |
| --- | --- | --- | --- | --- |
|  | Maternal Mortality |  |  |  |
|  | Maternal Health  Services |  |  |  |
| Hospitalization due sensitive causes | Hospitalization | Hospital* AND (ambulatory care sensitive* OR Primary care sensitive*) | Hospitalização | Hospitalização  Hospitalização por condições sensíveis a atenção primária  Internações por condições sensíveis à atenção primária  Hospitaliza$ or Interna$ AND ( condições sensíveis OR condição sensivel) |
|  |  |  |  |  |

# Search strategy

## Medline

-Population

1. Brazil[MeSH Terms]

2. Brazil*[Title/Abstract]

**3. #1 OR #2**

-Intervention

4. Family Practice[MeSH Terms] OR Primary Health Care[MeSH Terms]

5. Family health program[Title/Abstract] OR Family health strategy[Title/Abstract] OR Primary health care[Title/Abstract] OR Family health progra*[Title/Abstract] OR Family health strateg*[Title/Abstract] OR Family Practice[Title/Abstract]

**6. #4 OR #5**

-Outcomes

7. Tuberculosis[MeSH Terms] OR Mycobacterium tuberculosis[MeSH Terms] OR Latent Tuberculosis[MeSH Terms]

8. Tuberculosis[Title/Abstract] OR LTBI[Title/Abstract] OR Latent Tuberculosis[Title/Abstract] OR Mycobacterium tuberculosis[Title/Abstract]

9. Tuberc*[Title/Abstract] AND (Latent[Title/Abstract] OR infection[Title/Abstract])

**10. #7 OR #8 OR #9**

11 Child Nutrition Disorders[MeSH Terms]

12. Child Nutrition Disorders[Title/Abstract]

13. Child*[Title/Abstract] AND (Malnutrition[Title/Abstract] OR Nutrition*[Title/Abstract])

**14. #11 OR #12 OR #13**

15. (Infant Mortality[MeSH Terms]) OR Child Mortality[MeSH Terms]

16. (Child*[Title/Abstract] OR Infan*[Title/Abstract]) AND (morta*[Title/Abstract] OR death[Title/Abstract])

17. (Morta*[Title/Abstract] OR death [Title/Abstract]) AND (postneonatal[Title/Abstract] OR neonatal[Title/Abstract])

**18. #15 OR #16 OR #17**

19. Infant, Low Birth Weight[MeSH Terms]

20. ((Low[Title/Abstract] AND Birth[Title/Abstract] AND Weight[Title/Abstract]) AND Infan*[Title/Abstract]) OR Low Birth Weight*[Title/Abstract]

**21. #19 OR #20**

22. Diarrhea, Infantile[MeSH Terms]

23. (Diarrhea[MeSH Terms] OR Intestinal Diseases, Parasitic[MeSH Terms] OR Gastroenteritis[MeSH Terms]) AND Infant[MeSH Terms]

24. (Child*[Title/Abstract] OR infan*[Title/Abstract]) AND (Diarrhea[Title/Abstract] OR Gastroenteritis[Title/Abstract] OR Intestinal Parasito*[Title/Abstract])

**25. #22 OR #23 OR #24**

26. Breast Feeding[MeSH Terms]

27. Breast Feed* [Title/Abstract] OR Breastfeed*[Title/Abstract]

**28. #26 OR #27**

29. (Immunization Programs[MeSH Terms] OR Vaccination[MeSH Terms] OR Vaccines[MeSH Terms]

30. Vaccine*[Title/Abstract] OR immunization*[Title/Abstract]OR vaccination[Title/Abstract] or vaccina*[Title/Abstract]

**31. #29 OR #30**

32. Acquired Immunodeficiency Syndrome[MeSH Terms] OR HIV[MeSH Terms] OR Sexually Transmitted Diseases[MeSH Terms]

33. AIDS[Title/Abstract] OR STD[Title/Abstract] OR Sexually Transmitted[Title/Abstract] OR STDs[Title/Abstract] OR Acquired Immunodeficiency Syndrome[Title/Abstract]

34. Sexual*[Title/Abstract] AND transmi*[Title/Abstract]

**35. #32 OR #33 OR #34**

36. Neglected Diseases[MeSH Terms] OR Leprosy[MeSH Terms] OR Dengue[MeSH Terms]

37. Neglected Diseases*[Title/Abstract] OR Neglected Diseases*[Title/Abstract]) OR Lepra*[Title/Abstract] OR Hansen's disease[Title/Abstract] OR Dengue[Title/Abstract] OR Leprosy [Title/Abstract]

**38. #36 OR #37**

39. ((Prenatal Care[MeSH Terms]) OR Maternal Mortality[MeSH Terms]) OR Maternal Health Services[MeSH Terms]

40. (Prenatal Care[Title/Abstract]) OR Prenatal*[Title/Abstract]

41. Matern*[Title/Abstract] AND (Morta*[Title/Abstract] OR death [Title/Abstract] OR Health[Title/Abstract] OR service*[Title/Abstract])

**42. #39 OR #40 OR #41**

43. Hospitalization[MeSH Terms]

44. Hospital*[Title/Abstract] AND (ambulatory care sensitive*[Title/Abstract] OR Primary care sensitive*[Title/Abstract] AND ambulatory care*) OR Hospitalization[Title/Abstract]

**45. #43 OR #44**

- Combining

46. **#10 OR #14 OR #18 OR #21 OR #25 OR 28 OR #31 OR #35 OR #38 OR #42 OR #45**

47. **#3 AND #6 AND #46** /

48.  Publication date from 1994/01/01

## Web of Science

-Population

1. TS=Brazil*

-Intervention

2. TS= ("Family health program" OR "Family health strategy" OR "Primary health care" OR "Family health progra*" OR "Family health strateg*" OR "Family Practice")

-Outcomes

3. TS=(Tuberculosis OR LTBI OR Latent Tuberculosis OR "Mycobacterium tuberculosis")

4. TS= (Tuberc* SAME (Latent OR infection))

**5. #3 OR #4**

6. TS="Child Nutrition Disorders"

7. TS=(Child* SAME (Malnutrition OR Nutrition*))

**8. #6 OR #7**

9. TS=((Child* OR Infan*) SAME (morta* OR death))

10. TS= ((Morta* OR death) SAME (postneonatal OR neonatal))

**11. #9 OR #10**

12. TS= ((Low AND Birth AND Weight ) SAME Infan*)

13. TS= "Low Birth Weight*"

**14. #13 OR #12**

15. TS= ((Child* OR infan*) AND (Diarrhea OR Gastroenteritis OR Intestinal Parasito*))

16. TS= ("Breast Feed*" OR Breastfeed*)

17. TS= (Vaccine* OR immunization*OR vaccination OR vaccina*)

18. TS= (AIDS OR STD OR Sexually Transmitted OR STDs OR (Acquired SAME Immunodeficiency SAME Syndrome))

19. TS= (Sexual* SAME transmi*)

**20. #18 OR #19**

21. TS= (Neglected Diseases* OR Neglected Diseases* OR Lepra* OR Hansen's disease OR Dengue OR Leprosy)

22. TS= (Prenatal Care OR Prenatal*)

23. TS= (Matern* AND (Morta* OR death OR Health OR service*))

**24. #22 OR #23**

25. TS= Hospitalization

26. TS= (Hospital* AND (ambulatory care sensitive* OR Primary care sensitive* OR "primary care" ambulatory care*))

**27. #25 OR #26**

Combining

28**. #5 OR #8 OR #11 OR #14 OR #15 OR #16 OR #17 OR #20 OR #21 OR #24 OR #27**

29**. #1 AND #2 AND #28**

30. Timespan=1994-2016

## Lilacs

Population

(Brasil OR Brasil$ OR Brazil OR Brazil$)

Intervention

(PSF$ OR ESF$ "Saúde da Família" OR "Programa Saúde da Família" OR "Programa de Saúde da Família" OR "Estratégia de Saúde da Família" OR "Estratégia de Saúde na Família" OR "Medicina de Família e Comunidade" OR "Estratégia Saúde da Família" OR "Atenção Básica" OR "Atenção Básica à Saúde" OR "Atenção Primária de Saúde")

Outcomes

((("Tuberculose" OR "Tuberculose Latente" OR "Infecção Tuberculosa Latente" OR ILTB$) OR (Tuberc$ AND (latent$ OR infec$)))

(("Transtornos da Nutrição Infantil"  OR "Desnutrição Infantil" OR "Desnutrição em Crianças" OR "Desnutrição da Criança") OR (Desnutri$ AND (Criança$ OR infantil)))

(("Mortalidade infantil" OR "Mortalidade Pós-neonatal" OR "Mortalidade Neonatal" OR "Transtornos da Nutrição Infantil" OR "Mortalidade da Criança" OR "Mortalidade de Crianças" OR "Mortalidade na Infância") OR (Morta$ AND (neonatal OR infa$ OR Pós-neonatal OR Criança)))

(("Aleitamento materno") OR (aleitamento AND materno AND exclusivo))

((Diarreia OR Gastroenterite OR Disenteria AND (infan$ OR criança)) OR ("Diarreia Infantil"))

("Programas de Vacinação" OR "Programas de Imunização"  OR "Vacinação" OR "Programa Nacional de Imunizações" OR "Calendário Básico de Vacinação da Criança" OR " Calendário de Vacinações" OR "Calendário Nacional de Vacinação" OR Vacina$ OR Imunização)

(SIDA OR AIDS OR "Síndrome da Imunodeficiência Adquirida" OR "Síndrome de OR "Imunodeficiencia Adquirida " OR HIV OR "Doenças Sexualmente Transmitidas" OR "Doenças Sexualmente Transmissíveis" OR "Doenças de Transmissão Sexual" OR DST$)

("Doenças Esquecidas" OR "Doença Negligenciada"  OR "Doenças Negligenciadas" OR "Febre da dengue" OR Dengue OR Lepra$ OR Hanseniase OR "Doença de Hansen")

(("Assistência Pré-Natal" OR "Cuidado Pré-Natal" OR Pré-Natal$ OR "Mortalidade Materna") OR (Mater$ AND Mort$))

(("Recém-Nascido de Baixo Peso" OR "Baixo Peso ao Nascer ") OR (Nasc$ AND (Baixo AND Peso)))

(("Hospitalização" OR "Hospitalização por condições sensíveis a atenção primária" OR "Internações por condições sensíveis à atenção primária") OR (Hospitaliza$ OR Interna$ AND ( condições sensíveis OR condição sensivel))))

Combining

(Brasil OR Brasil$ OR Brazil OR Brazil$)

AND

(PSF$ OR ESF$ OR "Saúde da Família" OR "Programa Saúde da Família" OR "Programa de Saúde da Família" OR "Estratégia de Saúde da Família" OR "Estratégia de Saúde na Família" OR "Medicina de Família e Comunidade" OR "Estratégia Saúde da Família" OR "Atenção Básica" OR "Atenção Básica à Saúde" OR

"Atenção Primária de Saúde")

AND

(("Tuberculose" OR "Tuberculose Latente" OR "Infecção Tuberculosa Latente" OR ILTB$ OR "Transtornos da Nutrição Infantil"  OR "Desnutrição Infantil" OR "Desnutrição em Crianças" OR "Desnutrição da Criança" OR "Mortalidade infantil" OR "Mortalidade Pós-neonatal" OR "Mortalidade Neonatal"   OR "Transtornos da Nutrição Infantil" OR "Mortalidade da Criança" OR "Mortalidade de Crianças" OR "Mortalidade na Infância" OR "Aleitamento materno" OR "Diarreia Infantil" OR "Programas de Vacinação" OR "Programas de Imunização"  OR "Vacinação" OR "Programa Nacional de Imunizações" OR "Calendário Básico de Vacinação da Criança" OR " Calendário de Vacinações" OR "Calendário Nacional de Vacinação" OR Vacina$ OR Imunização OR SIDA OR AIDS OR "Síndrome da Imunodeficiência Adquirida" OR "Síndrome de Imunodeficiencia Adquirida " OR HIV OR "Doenças Sexualmente Transmitidas" OR "Doenças Sexualmente Transmissíveis" OR "Doenças de Transmissão Sexual" OR DST$ OR "Doenças Esquecidas" OR "Doença Negligenciada" OR "Doenças Negligenciadas" OR "Febre da dengue" OR Dengue OR Lepra$ OR Hanseniase OR "Doença de Hansen" OR "Assistência Pré-Natal" OR "Cuidado Pré-Natal" OR Pré-Natal$ OR "Mortalidade Materna" OR "Hospitalização" OR "Hospitalização por condições sensíveis a atenção primária" OR "Internações por condições sensíveis à atenção primária" OR "Recém-Nascido de Baixo Peso" OR "baixo Peso ao Nascer ") OR

((Tuberc$ AND (latent$ OR infec$)) OR (Desnutri$ AND (Criança$ OR infantil)) OR (aleitamento AND materno AND exclusivo) OR (Morta$ AND (neonatal OR infa$ OR "Pós-neonatal" OR Criança)) OR (infan$ AND (Diarreia OR Gastroenterite OR Disenteria)) OR (Nasc$ AND (Baixo AND Peso)) OR

(Hospitaliza$ AND ("condições sensíveis" OR "condição sensivel"))))

Years from 1994-2016
