# Supplementary material for: The impact of the Brazilian family health on selected primary care sensitive conditions: A systematic review
Source: PLoS One. 2017 Aug 7;12(8):e0182336. doi: 10.1371/journal.pone.0182336 (PMC5546674; doi:10.1371/journal.pone.0182336)
Supplement: S2 Table — (DOCX) [file pone.0182336.s004.docx]

**Supplementary Tables for**

**“**The Impact of the Brazilian Family Health on Selected Primary Care Sensitive Conditions: A Systematic Review.**"**

**Note that references are found in main text**

# Table S2- Summary of methods of included studies

| **Author, year** | **Setting** | **Age** | **Years of study** | **Type of data and Sources** | **Methods** | **Adjusted for** |
| --- | --- | --- | --- | --- | --- | --- |
| Aquino, 2009^23^ | 721 of 5561 municipalities | < 1 year | 1996-2004 | Secondary data:  -Mortality Information System (DATA-SUS/SIM)  -Information System on Live Births (DATA-SUS /SINASC)  -Primary Care Information System (DATA-SUS/SIAB)  - Department of Informatics of Brazilian System  Health  -Hospital Information System (DATA-SUS/ SIH/SUS)  -Brazilian Census- Brazilian Institute of Geography and Statistics [IBGE (91-2000)]  -Human Development Atlas | -Ecological study  -FHS coverage stratified into:  No FHS coverage,  Incipient coverage: <30.0%  Intermediate coverage:  30%-69.9% or > 70% for <4 years  Consolidated coverage: >70% for >4 years  -Used fixed – effects regression model  -Controlled for *Bolsa Família:* N/A | Socioeconomic indicators: *Per-capta* income, GINI Index, illiteracy, fertility rate  Sanitary condition: running water  Health access: Hospitalization rate per 1,000 habitants |
| Rasella, 2010^24^ | 2,601 municipalities | < 5 years (including analysis of neonatal, postnenonatal, child mortality) | 2000-2005 | Secondary data:  -Mortality Information System (DATA-SUS/SIM)  -Information System on Live Births (DATA-SUS /SINASC)  -Primary Care Information System (DATA-SUS/SIAB)  - Department of Informatics of Brazilian System  Health (DATA-SUS)  -Hospital Information System (DATA-SUS/ SIH/SUS)  -Brazilian Census- Brazilian Institute of Geography and Statistics IBGE (91-2000)  -Human Development Atlas | -Ecological study  -FHS coverage stratified into:  No FHS,  Low: <30.0%  Intermediate: 30.0%-69.9% High: >70.0%  **-**Used fixed-effects regression model  -Controlled for *Bolsa Família:* N/A | Socioeconomic indicators: *Per-capta* income, illiteracy, fertility rate  Sanitary condition: running water, households with sewerage  Health access:  Presence of local hospitals |
| Macinko, 2007^25^ | 558 micro-regions | < 1 year | 1999-2004 | Secondary data:  - Department of Informatics of Brazilian System  Health (DATA-SUS)  -Brazilian Census-IBGE (not clear year)  -Institute of Applied Economic Research (IPEA) | -Ecological study  -FHS coverage stratified into quintiles  -Used fixed-effects regression model  -Controlled for *Bolsa Família:* N/A | Socioeconomic indicators: Socioeconomic class  Mother with no formal education  Health Access:  Mother with no prenatal care  Number of physician  Number of hospital beds  Low birth weight |
| Shei, 2013^26^ | 5,506 municipalities | < 1 year | 1998-2008 | Secondary data:  -Department of Informatics of Brazilian System  Health (DATA-SUS)  -The Ministry of Social Development  -Brazilian Institute of Geography and Statistics (IBGE) | -Ecological study  -The effect of FHS was a secondary intervention; *Bolsa Família* is the main intervention.  -FHS coverage (not categorized)  -Used fixed-effects regression model  -Controlled for *Bolsa Família:* Yes | Socioeconomic indicators  Fertility;  Literacy; per capita income;  Sanitary condition:  Piped water, a sewage system, and electricity; Health Access  Supply, or the numbers of physicians per 1,000 habitants and nurses per 10,000 habitants |
| Serra, 2005^27^ | 110 municipalities from São Paulo State | < 1year | 1998-2001 | Secondary data:  -São Paulo Health Information  -Fundação Sistema Estadual de Análise de Dados – SAED  - Department of Informatics of Brazilian System  Health (DATA-SUS)  -Brazilian Institute of Geography and Statistics -IBGE  -Institute of Applied Economic Research (IPEA)  -Department of Informatics of Brazilian System  Health (DATA-SUS)  -Ministry of Education | -Ecological study  -FHS and PACS (community health worker program) coverage  -Used fixed-effect regression model  **-**Controlled for *Bolsa Família:* N/A | Socioeconomic indicators:, female schooling  Sanitary condition:  Total county expenditure with running water and sewerage  Health Access  Hospital beds and physicians per 1,000 habitants, |
| Rocha, 2013^28^ | 1,023 municipalities | < 1year | 1999-2003 | Secondary data:  -Brazilian Institute of Geography and  Statistics (IBGE)  -Brazilian Health Surveillance Agency (ANVISA)  -Department of Informatics of Brazilian System  Health (DATA-SUS) | -Ecological study  -FHS and PACS (community health worker program) coverage  -Difference in differences  **-**Controlled for *Bolsa Família:* No | Socioeconomic indicators: *Per-capta* income, number of elementary teachers with high level education per 1,000 habitants,  Sanitary condition  Deaths due infectious diseases per 1,0000 habitants  Health Access  Hospital beds per 1,000 habitants |
| **Author, year** | **Setting** | **Age** | **Years of study** | **Type of data and Sources** | **Methods** | **Adjusted for** |
| Guanais, 2009^29^ | 2,700 Municipalities | 28 days-1year | 1998-2006 | Secondary data:  -Department of Informatics of Brazilian System  Health (DATA-SUS)  -Ministry of Finance  -Institute for Applied Economic Research (IPEA)  -Brazilian Institute of Geography and Statistics (IBGE) | -Ecological study  -FHS coverage (not categorized)  -Used a two-way fixed effect regression  **-**Controlled for *Bolsa Família:* No | Socioeconomic indicators: illiteracy rate, *per capta* income, municipal size population  Sanitary condition: clean water supply  Health Access: ambulatory appointments |
| Guanais, 2013^30^ | 4,583 municipalities | 28 days-1year | 1998-2010 | Secondary data:  -The Ministry of Social Development  -Department of Informatics of Brazilian System  Health (DATA-SUS) | -Ecological study  -FHS coverage (not categorized)  -Fixed effect regression model  **-**Controlled for *Bolsa Família:* Yes | Socioeconomic indicators: Formal job, Fertility rate, schooling rate  Sanitary condition: clean water supply  Health Access: Hospital beds, prenatal consultation |
| Macinko, 2006^31^ | All 26 States and national capital city | <1year | 1990-2002 | Secondary data  -Primary Care Information System (DATA-SUS/SIAB)  -Brazilian Institute of Geography and Statistics (IBGE)  -Institute of Applied Economic Research (IPEA) | -Ecological study  -FHS coverage (not categorized)  -Used fixed-effects regression model  **-**Controlled for *Bolsa Família:* N/A | Socioeconomic indicators  Fertility; female illiteracy; per capita income;  Sanitary condition:  Piped water, a sewage system,  Health Access  Numbers of physicians and nurses per 10,000 habitants, number of hospital beds per 1,000 habitants. |
| Zanini, 2009^32^ | 35 Micro regions from Rio Grande do Sul State. | < 1 year | 1994-2004 | Secondary data  -Mortality Information System (DATA-SUS/SIM)  -Information System on Live Births (DATA-SUS /SINASC)  -Department of Informatics of Brazilian System  Health (DATA-SUS)  -Brazilian Institute of Geography and Statistics (IBGE)  -Primary Care Information System (DATA-SUS/SIAB)  -Institute of Applied Economic Research (IPEA)  -National Supplementary Health Care  System (ANS)  -Foundation for Economics and Statistics (FEE);  -National System of Urban Indicators (SNIU)  -Rio Grande do Sul State Socioeconomic Atlas, Census | -Ecological study  -FHS coverage (not categorized)  -Used random effects regression model  **-**Controlled for *Bolsa Família:* N/A | Socioeconomic indicators:  per capita household income less than half the minimum wage, illiteracy, mean schooling, socioeconomic development index, and household crowding  Sanitary condition:  Sewerage system  Health Access  Number of practicing physicians,  hospital beds and hospitals per 1,000 habitants |
| Rocha, 2010 ^33^ | 4,488 municipalities | < 5 years | 1995-2003 | Secondary data  -Department of Informatics of Brazilian System  Health (DATA-SUS)  -Brazilian Institute of Geography and Statistics (IBGE)  -Applied Economic Research (IPEA)  -Institute of Research on Education (Instituto Nacional de Pesquisa em Educação).  -Brazilian National Household Survey (PNAD) | -Ecological study  -Explored possible heterogeneity of FHS considering regions, causes of death and initial conditions (pre-existing trends)  -FHS coverage (not categorized)  -Used difference in difference model  -Controlled for *Bolsa Família:* N/A | Socioeconomic indicators:  Number of schools and teachers per capita.  Health Access  Health infrastructure (hospital beds and hospitals per capita), immunization rates |
| Roncalli, 2006^34^ | 2,144 children | < 1 year | Not very clear, but they used info from 2000 and 2004 to create a score. The interviews should be between 2004-2006 | Primary data:  -Community Health workers (CHWs) interviewed families. | -Cross-sectional study  The study was classified “Quasi-randomized community trial”  -FHS and/or Community Health Workers (CHW) coverage stratified in “yes” vs. “no  - Used hierarchical model, not clear which regression  -Controlled for *Bolsa Família:* N/A | Socioeconomic indicators: Income, unemployment, percent of population living with less than minimal wage, illiteracy  Sanitary condition:  Piped water, regular garbage pickups service |

| **Author, year** | **Setting** | **Age** | **Years of study** | **Type of data and Sources** | **Methods** | **Adjusted for** |
| --- | --- | --- | --- | --- | --- | --- |
| Rasella, 2013^35^ | 2,853 municipalities from Brazil | < 5 years | 2004-2009 | Secondary data  -Mortality Information System (DATA-SUS/SIM)  -Information System on Live Births (DATA-SUS /SINASC)  -Primary Care Information System (DATA-SUS/SIAB)  -Ministry of Social Development  -Brazilian Institute of Geography and Statistics (IBGE) | -Ecological study  -FHS coverage stratified into:  No FHS (0.0%), Incipient (<30%)  Intermediate (≥30%-69.9% or >=70% for < 4years), Consolidated (≥70% and implemented for at least 4 years). - Used fixed effects, conditional negative binomial model  -Controlled for *Bolsa Família:*Yes | Socioeconomic indicators:  Monthly income per person,  Fertility rate, illiteracy rate  Sanitary condition:  Inadequate water supply,  Sewers, and garbage collection  Health Access  Rates of admissions to hospital per 100 habitants |
| Rasella, 2013^36^ | 224 municipalities from Bahia State | < 5 year | 2005-2008 | Secondary data  -Mortality Information System (DATA-SUS/SIM)  -Information System on Live Births (DATA-SUS /SINASC)  -Primary Care Information System (DATA-SUS/SIAB)  -Brazilian Institute of Geography and Statistics (IBGE)  -Hospital Information System (DATA-SUS/ SIH/SUS) | -Ecological study.  -The main intervention is not FHS, it is other government program to supply clean water, and FHS was one of co-variables analyzed.  -FHS coverage stratified into:  High > 70%, Middle 0-70%  - Used fixed effects, conditional negative binomial model  - Controlled for *Bolsa Família:*No | Socioeconomic indicators:  *Per capta* income, illiteracy  Sanitary condition:  Households connected to piped water  Health Access  Number of practicing physicians |
| Dourado, 2011^37^ | Brazil, all 26 States and Capital city | <80 years | 1999-2007 | Secondary Data  -Hospital Information System (DATA-SUS/ SIH/SUS) | -Ecological study  -FHS coverage, classified into quintiles:  First (<19%) Second (19%–32%)  Third (33%–48%) Fourth (49%–64%)  Fifth (≥65%)  -Used fixed effects, conditional negative binomial model  -Controlled for *Bolsa Família:*No | Socioeconomic indicators:  Household *per capta* below the poverty, female illiteracy,  Sanitary condition:  Households connected to piped water  Health Access  Number of hospital beds per 10,000 habitants, % of population covered by private health insurance, average number of medical consultations |
| Macinko, 2010^38^ | 5,507 municipalities | All | 1999-2007 | Secondary Data  -Hospital Information System (DATA-SUS/ SIH/SUS)  -Brazilian Institute of Geography and Statistics (IBGE)  -Primary Care Information System (DATA-SUS/SIAB)  -Applied Economic Research (IPEA | -Ecological study  -FHS coverage, classified into quintiles  -Results presented in prevalence hospitalization ratio.  -Used fixed effects regression model  -Controlled for *Bolsa Família:*No | Socioeconomic indicators: Income *per capta,* female illiteracy rate  Sanitary condition  Clean water supply  Health Access  Number of beds |
| Rasella, 2014^39^ | 1,622 municipalities | 20-74 years | 2000-2009 | Secondary Data  -Hospital Information System (DATA-SUS/ SIH/SUS)  -Mortality Information System (DATA-SUS/SIM)  -Primary Care Information System (DATA-SUS/SIAB)  -Brazilian Institute of Geography and Statistics (IBGE) | -Ecological study  -FHS coverage classified into: 1-without FHS, 2-incipient (<30%), 3-intermediate (30%-69%), 4-consolidated (≥70%)  -Used fixed effects, conditional negative binomial model  -Controlled for *Bolsa Família:*No | Socioeconomic indicators  Per capta income, illiteracy  Sanitary condition  Adequate sewerage  Health Access:  Presence of local hospital beds, number physicians per 1000 inhabitants |
| Carvalho, 2015^40^ | 188 municipalities from Pernambuco State | < 5 years | 1999-2009 | Secondary Data  -Hospital Information System (DATA-SUS/ SIH/SUS)  -Primary Care Information System (DATA-SUS/SIAB)  -Institute of Applied Economic Research (IPEA)  -United Nations Children’s Fund (UNICEF) | -Ecological study  -FHS coverage classified into: Incipient (< 30%); Intermediate (≥ _70% and time since establishment < four years) or (< 70% and ≥ _30%); Consolidated (≥ _70% and time since establishment ≥ _four years)  -Used fixed effects regression model  -Controlled for *Bolsa Família:*No | Socioeconomic indicators:  Illiteracy rate in younger ≤ 15 years, human development index, child development index  Sanitary condition  Proportion of people with piped water  Health Access  Prenatal care visits, presence of hospitals in each municipality |
| Pazó, 2014^41^ | 78 municipalities from Espirito Santo state | All | 2010 | Secondary Data  -Hospital Information System (DATA-SUS/ SIH/SUS)  -Primary Care Information System (DATA-SUS/SIAB)  -Brazilian Institute of Geography and Statistics (IBGE) | -Ecological study  -Compared FHS coverage in different municipalities  -Used hierarchical model in the multiple regression  -Controlled for *Bolsa Família:*No | Socioeconomic indicators:  *Per capta* income, population receiving less than half minimal wage, GINI index, illiteracy  Sanitary condition  Adequate sewerage |

| **Author, year** | **Setting** | **Age** | **Years of study** | **Type of data and Sources** | **Methods** | **Adjusted for** |
| --- | --- | --- | --- | --- | --- | --- |
| Guanais, 2009^42^ | 2,248 municipalities | > 18 years | 1998-2002 | Secondary Data  -Hospital Information System (DATA-SUS/ SIH/SUS)  -Department of Informatics of Brazilian System  Health (DATA-SUS)  -Brazilian Institute of Geography and Statistics (IBGE) | -Ecological study  -FHS and PACS (CHW) coverage (not categorized)  -Used fixed effects regression model  -Controlled for *Bolsa Família:*N/A | Socioeconomic indicators:  Population per capita, illiteracy, population older than 60 years  Sanitary condition:  Clean water supply  Health Access  Ambulatory facilities and hospital beds per 1000 |
| Luz, 2010^43^ | 82 municipalities from central and 55 municipalities Southeast regions of Minas Gerais | > 60 year | 2001-2007 | Secondary Data  -Mortality Information System (DATA-SUS/SIM)  -Hospital Information System (DATA-SUS/ SIH/SUS)  -Primary Care Information System (DATA-SUS/SIAB) | -Ecological study  -FHS coverage (not categorized)  -Poisson regression models were used  -Only included cities >5,000 habitants, did not specify the analysis of missing data, but weighted by population size  -Controlled for *Bolsa Família:* No | Health Access  Presence of emergency system, ambulances care. |
| Macinko, 2011^44^ | 558 microregions | 20-79 years | 1999-2007 | Secondary Data  -Hospital Information System (DATA-SUS/ SIH/SUS)  -Primary Care Information System (DATA-SUS/SIAB) | -Ecological study  -FHS coverage classified into  0-24%, 25-49% 50-74%, 75%-100%  -Used Fixed effect model  -Controlled for *Bolsa Família:* No | Socioeconomic indicators per capita income, illiteracy,  Sanitary condition  Clean water supply  Health Access  Annual mean number of doctor visits per capita and the proportion of individuals with private health insurance. |
| Mendonça, 2012^45^ | Belo Horizonte, MG, census tract, 1,909 | All | 2003-2006 | Secondary Data  -Hospital Information System (DATA-SUS/ SIH/SUS)  -Primary Care Information System (DATA-SUS/SIAB)  -Brazilian Institute of Geography and Statistics (IBGE) | -Ecological study  -FHS coverage (not categorized)  -Used random effect model  -Controlled for *Bolsa Família:* No | Socioeconomic indicators  Vulnerability index  Health Access  Physician consultations/inhabitant/year |
| Monahan, 2013^46^ | 12 (of 47 existing) municipalities in Bahia state | < 5 years | 1999-2007 | Secondary Data  -Hospital Information System (DATA-SUS/ SIH/SUS)  -Primary Care Information System (DATA-SUS/SIAB)  -Brazilian Institute of Geography and Statistics (IBGE)  -Applied Economic Research (IPEA) | -Ecological study  -FHS coverage (not categorized)  -Used fixed effect model  -Controlled for *Bolsa Família:* No | Socioeconomic indicators: human development index (HDI), illiteracy  Health Access: number of physicians per 10,000 |
| Silva, 2011^47^ | Juiz de For a, MG, not ecological | 1,058 patients | 2008 | Secondary Data  -Hospital Information System (DATA-SUS/ SIH/SUS)  - Brazilian Institute of Geography and Statistics (IBGE)  -National Records of institute of health (CNES) | -Cross-sectional study  -Compared FHS vs. traditional primary care unit  -Multi-level regression  -Controlled for *Bolsa Família:* No | Socioeconomic indicators:  human development index (HDI) |
| Mendoza, 2011^48^ | Rio Grande, RS , 961 participants | Not Specified | 2007 | Primary Data,  Questionnaires, mother of children over 500g or 20 weeks were interviewed in 2 hospitals, until 24 hours after birth delivery. | -Cross-sectional study  -Compared FHS vs. traditional primary care units  -Used Poisson regression  -Controlled for *Bolsa Família:* No | Socioeconomic indicators  Income  Health Access:  Number of physician appointments |
| Lima-Costa, 2013^49^ | Belo Horizonte, MG, 7,534 participants | >60 years for flu vaccination | 2010 | Primary data  Complementary health questionnaire to the *Pesquisa de Emprego e Desemprego da Região Metropolitana de BH.* This was based in 5798 (77.3%) of the 7500 households of the region. | -Cross-sectional study  -4 groups of comparison:  1)Traditional primary care unit (PCU) and does not have access to other type of care  2)Private health insurance  3)Lives in area with FHS, but does not use FHS and also does not have private health insurance,  4)Lives in area covered by FHS and use the services.  -Used Poisson regression  -Controlled for *Bolsa Família:* No | Socioeconomic indicators:  Schooling and socioeconomic class |

| **Author, year** | **Setting** | **Age** | **Years of study** | **Type of data and Sources** | **Methods** | **Adjusted for** |
| --- | --- | --- | --- | --- | --- | --- |
| Nery, 2014^50^ | 1,358 municipalities | Not specified | 2004-2011 | Secondary Data:  -Surveillance for diseases notification (DATA-SUS/SINAN)  -Primary Care Information System (DATA-SUS/SIAB)  -Ministry of Social Development  -Brazilian Institute of Geography and Statistics (IBGE) | -Ecological study  -FHS coverage was categorized according to tertiles of coverage (1^st^ tertile: 0–72.02%, 2^nd^ tertile: 72.03– 95.06% and 3^rd^ tertile: over 95.06%)  -Used Fixed effect model  -Controlled for *Bolsa Família:* Yes | Socioeconomic indicators:  Illiteracy, unemployment rate, households crowding, GINI index, *pe rcapta* income, % of population living below poverty  Sanitary condition  Urbanization rate |
| Albuquerque, 2007 ^51^ | 1,396 participants | Not specified | 2001-20013 | Primary Data:  Interviewed patients in primary care units | -Cross sectional study  -FHS vs. traditional primary care unit  -Controlled for *Bolsa Família:* N/A | Socioeconomic indicators:  Illiteracy and alcohol consumption |
| Benício, 2013^52^ | 3,931 children | 6 months-5 years | 2006-2007 | Secondary Data  -Brazilian National Household Survey (PNAD)  -National Surveillance in Nutritional and Health  -Primary Care Information System (DATA-SUS/SIAB) | -Cross sectional study  -Malnutrition was accessed using Z- score for high (<-2), by age.  -Percent of FHS coverage (5 categories: <15%, 15-30%, 30-50%, 50%-70%, > 70%)  -Used logistic regression  -Controlled for *Bolsa Família:* No | Socioeconomic indicators:  Socioeconomic status, household crowding  Sanitary condition  Clean water |
| Araújo, 2012^53^ | 897 Brazilian municipalities with at least 1 case of congenital syphilis reported | Newborns | 2003-2008 | Secondary data  -Surveillance for diseases notification (DATA-SUS/SINAN)  -Hospital Information System (DATA-SUS/ SIH/SUS)  -Mortality Information System (DATA-SUS/SIM) | -Ecologic d study.  -Municipalities were the unit of analysis.  -FHS coverage (not categorized)  -Used logistic regression analysis  - Controlled for *Bolsa Família:* No | Health Access:  Prenatal coverage |

**Abbreviations**

-ANS: Supplementary Health Care System

**-**Brazilian Health Surveillance Agency (ANVISA)

-Brazilian National Household Survey (PNAD)

-CI: Confidential intervals

-CNES: National Records of institute of health

-Data SUS: Department of Informatics of Brazilian Unified Health System

-DATA-SUS /SINASC: Information System on Live Births

-DATA-SUS/SIAB: Primary Care Information System

-DATA-SUS/ SIH/SUS: Hospital information system

-DATA-SUS/SIM: Mortality Information System

-DATA-SUS/SINAN: Surveillance for diseases notification

-FHS: Family Health Strategy

-IBGE- Brazilian Institute of Geography and Statistics

-IPEA Institute of Applied Economic Research

-N/A: Not applicable

-SUS: from Portuguse, Sistema Único de Saúde (Unified Health System)

-SNIU: National System of Urban Indicators

-PACS: *Programa de Agente Comunitário de Saúde*

*-*PCU: Primary Care Unit

-UNICEF:United Nations Children’s Fund
