# Supplementary material for: The impact of the Brazilian family health on selected primary care sensitive conditions: A systematic review
Source: PLoS One. 2017 Aug 7;12(8):e0182336. doi: 10.1371/journal.pone.0182336 (PMC5546674; doi:10.1371/journal.pone.0182336)
Supplement: S3 Table — (DOCX) [file pone.0182336.s005.docx]

**Supplementary Tables for**

**“**The Impact of the Brazilian Family Health on Selected Primary Care Sensitive Conditions: A Systematic Review.**"**

**Note that references are found in main text**

# Table S3- Descriptive results of included studies about neonatal (up to 27 days), post-neonatal (28 days- 1 year), infant (neonatal + post-neonatal) and child (up to 5 years) mortality

| **Author, year** | **Outcome Studied** | **Measure of effect** | **Main results** | **Interpretation of main results** | **Quality assessment** |
| --- | --- | --- | --- | --- | --- |
| Aquino, 2009^23^ | Neonatal and post-neonatal mortality | Mortality rate ratio (95% CI) | Neonatal mortality rate ratio  - No FHS coverage: 1.00  - <30% coverage: 0.90 (0.89;0.92)  - 30-69.9% coverage or > 70% for <4 years: 0.86 (0.84;0.89)  - >70% for >4 years coverage: 0.81 (0.76;0.88)  Post-neonatal mortality rate ratio  - No FHS coverage: 1.0  - <30% coverage: 0.82 (0.8;0.84)  - 30-69.9% coverage (or > 70% for <4 years): 0.78 (0.75;0.81)  - >70% for >4 years coverage: 0.69 (0.62;0.76) | There was a significant reduction of neonatal (up to 19% in cities with consolidated FHS) and post-neonatal (up to 31% in cities with consolidated FHS) mortality with increasing FHS coverage. The effect of infant mortality was higher when baseline infant mortality was evaluated: 34% of reduction in cities with the highest infant mortality (>24.1/1000) and higher coverage. | Score: 43  High quality |
| Rasella, 2010^24^ | Neonatal and post- neonatal mortality | Mortality rate ratio (95% CI) | Neonatal mortality rate ratio:  - No FHS: 1.00  - <30% coverage: 0.97 (0.95;1.00)  - 30.0%-69.9%: 0.92 (0.89;0.95)  - >70.0%: 0.89 (0.86;0.93)  Post-neonatal mortality rate ratio:  - No FHS: 1.00  - <30% coverage: 0.93 (0.90;0.97)  - 30%-69.9% coverage: 0.88 (0.84;0.91)  - >70% coverage: 0.83 (0.78;0.87) | There was a significant reduction of neonatal (up to 11% in cities with high FHS coverage) and post-neonatal (up to 17% in cities with high FHS coverage) mortality with increasing FHS coverage. | Score:43  High quality |
| Macinko, 2007^25^ | Neonatal and post-neonatal mortality | Percent change in post-neonatal mortality with the increase unit change in FHS coverage (slope of regression, and 95% CI) | Percent change in post- neonatal mortality rate  FHS  - Slope: -0.0591 (-0.909;-0.273)  Large urban populations:  - Slope: -0,2873 (-4,154;-1,593)  Mothers with no prenatal care  - Slope +0,0784 (0.035;0.958) | The FHS had no effect in neonatal mortality. Each 10% increase of the FHS coverage was associated with around a 0.6% decrease in the post-neonatal period. Each 10% increase of mothers with no prenatal care was associated with a 0.8% increase in the post-neonatal mortality rate. | Score:42  High quality |
| Shei, 2013^26^ | Neonatal and post-neonatal mortality | Change in neonatal and post-neonatal mortality per 1000 live births with the increase unit change in FHS coverage (slope of regression, p-value). Municipalities were categorizing in quintile according the baseline mortality rate, the 5^th^ had the highest infant mortality rate baseline. | Post- neonatal mortality rate per 1000 live births: (municipalities per quintile of baseline rates)  - Slope (p value):  1^st^: −0.0001 (p value NS)  2^nd:^ −0.004 (p value NS)  3^rd^: −0.009 (p value < 0.001)  4^th^: −0.001 (p value NS)  5^th:^ −0.012 (p value NS)  Interaction between *Bolsa Família* coverage and high Family Health Program coverage  -0.029 (p value <0.05) | There was a minimal decrease in post-neonatal mortality rate restricted to those with median baseline mortality rate (0.09 fewer post-neonatal deaths per 1000 live births for each 10% increase in the FHS coverage in the 3^rd^ quintile). Calculated decrease in post-neonatal mortality rate= 1.4% per 10% increase in FHS. * There was no effect in neonatal mortality. Interaction between BFP and FHS: Each 10% of BFP coverage in areas with high coverage of FHP (>70% of coverage) was associated 0.3 fewer deaths per 1,000 habitants. | Score:35  Low Quality |
| Serra, 2005^27^ | Neonatal and post-neonatal mortality | Percent change in neonatal and post-neonatal mortality with the increase unit change in FHS coverage (slope of regression, p- value) | Neonatal mortality rate per 100 live births:  - Slope: -0.008 (p value < 0.05)  Post-neonatal mortality rate per 100 live births:  - Slope: -0.001 (p value NS) | The FHS had no effect in post-neonatal mortality. Regarding neonatal mortality, each 10% increase of the FHS coverage in areas with high coverage was associated with around 0.8 fewer deaths per 1000 live births. | Score:39  High quality |
| Rocha, 2013^28^ | Neonatal and post-neonatal mortality | Difference in Difference in the neonatal and post neonatal mortality. Coverage was defined as presence or not of FHS in the municipality. | Neonatal mortality per 1000 live births:  Difference in mortality rate ranged from -0.781 (year 2000) to -1.383 (year 2003), p value<0.01  Post-neonatal mortality per 1000 live births:  Difference in mortality rate ranged from -0.121 (year 2000) to -0.987 (year 2003) p value<0.01 | FHS had higher impacts on infant mortality (including neonatal and post-neonatal mortality) in all cities; the impact was higher in cities without hospitals (data not shown in this table). Reduction in the neonatal mortality ranged from 7.8% to 13.8%. Reduction in post-neonatal: 1.2%-9.8% | Score:38  High quality |

#

| **Author, year** | **Outcome Studied** | **Measure of effect** | **Main results** | **Interpretation of main results** | **Quality assessment** |
| --- | --- | --- | --- | --- | --- |
| Guanais, 2009^29^ | Post-neonatal mortality | Change in post-neonatal mortality per 1000 live births with the increase unit change in FHS coverage (slope of regression, 95% CI) | Post- neonatal mortality rate per 1000 live births  FHS coverage >75%:  - Slope: -0.86 (-1.31;-0.42)  Ambulatory care (any kind of primary care)  - Slope: -0.60 (-1.18; -0.03) | Areas with high coverage (>75%) had fewer 0.9 deaths per 1000 live births. Calculated decrease in post-neonatal mortality rate= 6% per 10% increase in FHS. * | Score:45  High quality |
| Guanais, 2013^30^ | Post-neonatal mortality | Predicted change in mortality rate per 1000 live births (95% CI), with the increase of FHS coverage and BFP coverage | Predictive Post-neonatal mortality rate per 1000 live births:  No FHS coverage and 25% of BFP coverage:  5.24 (4.95; 5.53)  100% coverage of FHS and 25% of BF coverage:  3.54 (2.77;4.31)  No FHS coverage and 60% BFP coverage:  4.65 (4.36;4.94)  100% FHS coverage and 60% BFP coverage:  1.38 (0.88;1.89) | There was a significant reduction of post-neonatal mortality with increasing FHS coverage. The effect was independent of *Bolsa Família* (BFP), although its magnitude was very reduced when BFP was added to the model. Calculated decrease in post-neonatal mortality rate= 66% considering a 100% FHS coverage. * | Score:41  High quality |
| Macinko, 2006^31^ | Infant mortality | Percentage change in mortality rate when an increase of 10% in the FHS coverage (slope of regression, 95% CI) | Percentage change of infant mortality rate  FHS  - Slope -4.56 (-5.68;-3.44)  Female illiteracy  -Slope +16.82 (11.38;22.26) | Each 10% increase of the FHS coverage is associated with a reduction of 4.56% of the infant mortality rate. | Score:44  High quality |
| Zanini, 2009^32^ | Infant mortality | Change in infant mortality per 1000 live births with the increase unit change in FHS coverage (slope of regression, standard error) | Infant mortality per 1000 live births  FHS  - Slope: -0.10 (SE 0.03)  Poverty (% population with per capita income < ½ minimum wage)  - Slope: -0.21 (SE 0.04) | Each 10% increase of the FHS coverage is associated with 1 fewer death per 1000 live births. Calculated decrease in infant mortality rate= 5% per 10% increase in FHS. * | Score:41  High quality |
| Rocha, 2010^33^ | Infant mortality | Change in infant mortality per 1000 live births with the increase unit change in FHS – the unit was the additional years in the FHS | Infant mortality per 1000 live births  First year in the FHS: slope -0.5690 (SE 0.2701)  Eighth year in the FHS: slope -5.408 (SE 1.5642) | Average mortality reduction was 0.69/1000 live births per additional year since FHS implementation. FHS reduced 0.6 deaths per 1000 live births in the first year of implementation and 5 deaths per 1000 live births in the 8^th^ year.Calculated decrease in infant mortality rate= 2.54% for each additional year of FHS coverage. * | Score:37  High quality |
| Roncalli, 2006†^34^ | Infant mortality | Mortality Ratio (95 % CI) | Proportions and Prevalence rate  No coverage: 6.1%, 1.00  CHWs only: 6.8%, 0.89 (0.35;2.27)  FHS only: 4.6%, 1.31 (0.55;3.08)  FHS+ CHWs: 5.2%, 1.17 (0.51;2.69) | No differences in infant mortality rates were found. | Score:41  High quality |
| Rasella, 2013^35^ | Child (< 5 years) mortality | Child Mortality Rate Ratio (95 % CI) | Child Mortality rate ratio  No FHS coverage: 1.00  <30% coverage: 0.99 (0.94;1.04)  30%-70% coverage: 0.93 (0.88;0.97)  ≥ 70 and implemented ≥ 4 years: 0.88 (0.83;0.93) | There was a significant reduction of child mortality with increasing FHS coverage (up to 12% in cities with high FHS coverage); this effect was independent of the BFP effect.  BFP had the strongest effect, followed by FHS. Other variables had a modest effect (<10%) | Score:45  High quality |
| Rasella, 2010^24^ | Child (<5 years) mortality | Child Mortality Rate Ratio (95 % CI) | Child mortality rate ratio  No FHS coverage: 1.00  <30% coverage: 0.96 (0.94;0.98)  30%-69.9% coverage: 0.91 (0.88;0.93)  >70% coverage: 0.87 (0.85;0.90) | There was a significant reduction of child mortality (up to 13% in cities with high FHS coverage) with increasing FHS coverage. | Score:43  High quality |

| **Author, year** | **Outcome Studied** | **Measure of effect** | **Main results** | **Interpretation of main results** | **Quality assessment** |
| --- | --- | --- | --- | --- | --- |
| Rasella, 2013^36^ | Child (<5 years) mortality from diarrhea | Change in infant mortality per 1000 live births with the increase unit change in FHS coverage (slope and p-value) | Child mortality rate per 1000 live births  Slope: -0,0087 (p value 0,029) | The effect of high FHS coverage was marginally significant in reducing child mortality from diarrhea, but the effect was very strong. Calculated decrease in child mortality rate= 5% per 10% increase in FHS. * | Score:45  High quality |
| Rocha, 2010^33^ | Child (1-4 years) mortality | Change in infant mortality per 1000 live births with the increase unit change in FHS – the unit was the additional years in the FHS (slope and p-value) | Child mortality per 1000 live births  First year in the FHS**:** -0.032 (0.0156)  Eighth year in the FHS: -0.2560 (0.0562) | The average reduction of child mortality was 0.035/1000 for each additional year since FHS implementation. Calculated decrease in child mortality rate= 3.18% per additional year in FHS. * | Score: 37  High quality |

**Abbreviations:**

CHW: Community health works

CI: Confidence intervals

BFP: *Bolsa família* Program

FHS: Family Health Strategy

NS: Not significant

**Notes**

*We calculated the reduction in the mortality rate percentage based on the rate informed by authors during the study period and their presented outcomes whenever possible.

†All but reference 34 are ecological studies.
