# Supplementary material for: The impact of the Brazilian family health on selected primary care sensitive conditions: A systematic review
Source: PLoS One. 2017 Aug 7;12(8):e0182336. doi: 10.1371/journal.pone.0182336 (PMC5546674; doi:10.1371/journal.pone.0182336)
Supplement: S4 Table — (DOCX) [file pone.0182336.s006.docx]

**Supplementary Tables for**

**“**The Impact of the Brazilian Family Health on Selected Primary Care Sensitive Conditions: A Systematic Review.**"**

**Note that references are found in main text**

# Table S4 Descriptive results of included studies about hospitalization

| **Author, year** | **Outcome Studied** | **Measure of effect** | **Main results** | **Interpretation of main results** | **Quality assessment** |
| --- | --- | --- | --- | --- | --- |
| Dourado, 2011^37^ | Hospitalization due to primary care sensitive causes | Relative Risk (95 % CI) | Relative Risk:  < 19% coverage: 1.00  19%-33% coverage: 0.958 (0.944;0.972)  33%-48% coverage: 0.966 (0.949;0.984)  49%-64% coverage: 0.992 (0.972;1.013)  >65%: 0.952 (0.927–0.977) | A minimal decrease in hospitalization rates for primary care sensitive causes was observed (up to 4.8% in areas with >65% coverage). | Score 42  High quality |
| Macinko, 2010^38^ | Hospitalization due to primary care sensitive causes, chronic diseases: asthma, stroke other cardiovascular vascular diseases, diabetes, chronic obstructive pulmonary diseases | Hospitalization rate ratios (p values) | Rate ratios for all chronic condition  Quintile 1 FHS coverage: 1.00  Quintile 2 FHS coverage: 0.96  Quintile 3 FHS coverage: 0.92  Quintile 4 FHS coverage: 0.89  Quintile 5 FHS coverage: 0.87  All p< 0.001 | The reduction of the hospitalization rate in the highest quintile coverage was 13% for all chronic diseases. For diabetes, the effect was the opposite: the highest coverage had the highest rate of hospitalization 9 % | Score: 41  High quality |
| Rasella, 2014^39^ | Hospitalization due to primary care sensitive causes (heart and  cerebrovascular diseases) | Hospitalization rate ratios  (95 % CI) | Rate ratios  Heart diseases  No FHS coverage: 1.00  >0%-30% coverage: 0.89 (0.87;0.91)  ≥30%-70% coverage: 0.83 (0.80;0.85)  ≥70% coverage: 0.70 (0.67;0.72)  Cerebrovascular diseases  No FHS coverage: 1  >0%-30% coverage: 0.88 (0.85;0.90)  ≥30%-70% coverage: 0.82 (0.79;0.85)  ≥70% coverage: 0.72 (0.69;0.75) | FHS reduced the rates of hospitalization for heart diseases by up to 30% and for cerebrovascular diseases by up to 28% in areas with the highest coverage. | Score: 45  High quality |
| Carvalho, 2015^40^ | Hospitalization due to primary care sensitive causes for children < 5 years | Hospitalization rate ratios  (95 % CI) | Rate ratios  < 30% coverage: 1.00  ≥30-70% or ≥ 70% coverage and < 4years implemented: 0.96 (0.93; 0.99)  ≥ 70% and > 4 years implemented: 0.94 (0.89;0.99) | The rates of hospitalization in municipalities with intermediate and consolidated FHS coverage were, respectively, 4% and 6% lower than those with incipient cov­erage. | Score:43  High quality |
| Roncalli, 2006^34^† | Hospitalization due to primary care sensitive causes (for diarrhea, lower respiratory infection in children) | Prevalence ratios  (95 % CI) | Proportions and Prevalence rate  Admissions for respiratory lower infection  No coverage: (23%) 1.00  CHWs only: (16%) 1.44 (1.05;2.00)  FHS only: (20%) 1.17 (0,90;1.54)  FHS + CHWs: (19%) 1.24 (0.95;1.61)  Admissions for diarrhea  No coverage: (19%) 1.00  Only CHWs: (11%) 1.76 (1.22;2.56)  Only FHS: (11%) 1.77 (1.30;2.41)  FHS + CHWs: (11%) 1.77 (1.31;2.39) | FHS reduced the rates of hospitalization for diarrhea by 77% but had no effect in the rates of hospitalization for respiratory infection. | Score:41  High quality |
| Pazó, 2014^41^ | Hospitalization due to primary care sensitive causes | Relative risk (95 % CI) | Relative Risk FHS vs. no coverage (reference)  Univariate analysis  RR 1.01 (1.01; 1.01)  Multivariate analysis  Relative risk not shown but p-value NS | The FHS had no effect on hospitalizations due to primary-care sensitive conditions. | Score:35  Low Quality |

| **Author, year** | **Outcome Studied** | **Measure of effect** | **Main results** | **Interpretation of main results** | **Quality assessment** |
| --- | --- | --- | --- | --- | --- |
| Rasella, 2013^36^ | Hospitalization due to diarrhea | Change in hospitalization rate per 10,000 children < 5 years with the increase unit change in FHS coverage in FHS coverage (slope and p-value) | Hospitalization Rates per 10,000 habitants  -Slope: + 0.0010 p=0.182 | The FHS was not significantly associated with hospitalization due to diarrhea. | Score:41  High quality |
| Guanais, 2009^42^ | Hospitalization due to primary care sensitive causes: circulatory conditions (hypertensive diseases, ischemic  heart diseases, and heart failure); diabetes  mellitus; and respiratory conditions  (asthma, bronchitis, and pneumonia) | Change in hospitalization rate per 10,000 habitants with the increase unit change in FHS coverage. | Women  Circulatory hospitalizations per 10,000  FHS: – 0.01 SE (0.02) p value NS  Only CHW: -3.15 SE (1.22) p-value <0.05  Diabetes mellitus hospitalizations per 10,000  FSH -0.02 (0.01) p-value<0.01  Only CHW -0.21 (0.34) p-value NS  Respiratory hospitalizations per 10,000  FHS -0.06 (SE 0.02) p-value <0.01  Only CHW -2,10 (1.27) p-value NS | FHS was significantly associated with reduction in hospitalization rates for diabetes mellitus (each 10% increase of FHS was associated with 0.2/10,000 reduction). Calculated decrease in hospitalization rate= 1.4% per 10% increase in FHS for diabetes and 0.71 for respiratory conditions. *  Effects were only shown for women; no effect could be shown for men. | Score:45  High quality |
| Luz, 2010^43^ | Hospitalization due to stroke and myocardium infarction | Percent change in hospitalization rate with the increase unit change in FHS coverage (slope and p value) | Women hospital admission due to stroke:  - Slope: 0.0008 (SE 0.0005) p-value NS  Women Hospital Admission due to myocardial infarction  - Slope: -0.0110 (SE 0.0020) p-value <0.05  Men hospital admission due to stroke  - Slope: 0.0004 (SE 0.0005) p-value NS  Men hospital admission due to myocardial  - Slope: -0.0002 (SE 0.0012) p-value NS | Each 10% of FHS was associated with a decrease of 11% in hospital admissions due to myocardium infarction among women. No association of FHS was observed in hospitalizations due to stroke in women. No effect in men. | Score:34  Low Quality |
| Macinko, 2011^44^ | Hospitalization due to primary care sensitive causes (not specified each condition) | Change in hospitalization rates per 10000 habitants with the increase unit change in FHS coverage (slope and 95% CI) | Hospitalization Rates per 10000 habitants  -Slope (CI 95%)  0%-24% coverage: 1.00  25-49% coverage: –0.35 (-3.95;3.26)  50-74% coverage: –3.46 (-8.10;1.17)  75%-100% coverage: –10.73 (-16.14;-5.33)  Only 75-100% was significant, p-value < 0.001 | There was a negative association between FHS coverage and hospitalization, i.e. the increase of coverage resulted in decreased hospitalization. This was only significant in the highest coverage strata. There was no effect in hospitalizations not due to primary care sensitive causes. | Score: 42  High quality |
| Mendonça, 2012^45^ | Hospitalization due to primary care sensitive causes (not specified) | Change in hospitalization rates per 10000 habitants with the increase unit change in FHS coverage – the unit was the additional months the FHS operation (slope and 95% CI) | Hospitalization Rates per 10000 Women  FHS  -Slope 1.0 (0.15;1.86)  Time of physician in the FHS team  -Slope -0.53 (-1.03; -0.04)  Hospitalization Rates per 10000 Men  FHS  -Slope 0.78 (0.10; 1.46)  Time of physician in the FHS team  -Slope -0.22 (-0.62;-0.16) | Between 2003 and 2006, there was a 17.9% reduction in hospitalization rates (versus 8.3% for hospitalizations due to non-primary care sensitive conditions). For each additional month of FHT operation, the number of hospitalizations increased by 1/10,000 inhabitants/year among women and by 0.7/10,000 inhabitants year among men (both p<0.001).  If same physician remained in the FHT, admissions were reduced by 0.5 and 0.2 respectively.  The effect of social vulnerability was stronger than the effect of FHS coverage. | Score:40  High quality |
| Monahan, 2013^46^ | Hospitalization due to primary care sensitive causes: gastroenteritis among children< 5y | Change in hospitalization rate with the increase unit change in FHS coverage (slope and standardized error) | Hospitalization Rats per 10000  Model 1 Adjusted for HDI  - Slope: -3.07 (SE 1.14) p-value <0.05  Model 2 Adjusted, for HDI, permanent sanitation structure, clean water availability, and garbage collection service.  -Slope: -1.83 (1.60) p-value NS | When relevant social variables are included in the model, no significant effect of the FHS coverage is observed on hospitalization rates. | Score:35  Low Quality |

| **Author, year** | **Outcome Studied** | **Measure of effect** | **Main results** | **Interpretation of main results** | **Quality assessment** |
| --- | --- | --- | --- | --- | --- |
| Silva, 2011†^47^ | Hospitalization due to primary care sensitive causes | Change in number of hospitalizations per unit change in FHS (slope and standardized error) | Number of hospitalization  FHS  Slope: -0.017 (0.106) p-value NS  Other models of Primary Care:  Slope: -0.085 (-0.108) p-value NS  Areas with any model of care (FHS + other):  +0.181 ( 0.109): Odds ratio 1.26 (1.03;1.43) | Areas with FHS or other models of primary care (any of both) had a 26% increase of hospitalization. There were no differences in hospitalization rates when either model was analysed separately. | Score:33  Low Quality |

**Abbreviations**

CHW: Community health works

CI: Confidence interval

BFP: *Bolsa família* Program

FHS: Family Health Strategy

HDI: Human development index

NS: Not significant

SE: Standard Error

**Notes**

*We calculated the reduction in the rate percentage based on the hospitalization rates informed by authors during the study period and their presented outcomes.

†All but reference 34 and 47 are ecological studies.
