# Supplementary material for: The impact of the Brazilian family health on selected primary care sensitive conditions: A systematic review
Source: PLoS One. 2017 Aug 7;12(8):e0182336. doi: 10.1371/journal.pone.0182336 (PMC5546674; doi:10.1371/journal.pone.0182336)
Supplement: S5 Table — (DOCX) [file pone.0182336.s007.docx]

**Supplementary Tables for**

**“**The Impact of the Brazilian Family Health on Selected Primary Care Sensitive Conditions: A Systematic Review.**"**

**Note that references are found in main text**

# Table S5 Descriptive results of included studies for all other health outcomes

| **Author, year** | **Outcome Studied** | **Measure of effect** | **Main Results** | **Interpretation of main results** | **Quality assessment** |
| --- | --- | --- | --- | --- | --- |
| Mendoza,2011†^48^ | Anti-tetanic vaccination, low-birth weight, perinatal mortality, admission of newborns to ICU, hospitalization during pregnancy, C-section | Prevalence ratio (95 % CI) | Proportions and Prevalence ratio for FHS over Traditional primary care unit  -C-section: 39.6% vs. 36.1%; 1.11 (0.89;1.38)  -Maternal admission: 18.8% vs. 9%; 2.0 (1.39;2.88)  -Tetanus vaccination during prenatal: 89.6% vs. 62.7%; 1.44 (1.29;1.61)  - Admission of newborns to ICU: 3.6% vs. 3.7%; 0.98 (0.58;1.90)  - Low weight newborn  8.9% vs. 9.8%; 0.87 (0.56;1.33) | Prenatal care in the FHS facilities resulted in a 44% increase in vaccination coverage but increased in 100% the rate of maternal admissions. No differences observed in the other health outcomes. | Score:31  Low Quality |
| Roncalli 2006†^34^ | Low-birth weight new born and Diphtheria, tetanus and pertussis vaccination completeness | Prevalence ratio (95 % CI) | Proportions and Prevalence rate  Low-birth weight new born:  No coverage: 12.1%; 1.00  CHW only: 11.1%; 1.10 (0.56;2.14)  FHS: 10.0%; 1.21 (0.67;2.17)  FHS + CHW: 10.3%; 1.18 (0.67;2.09)  Diphtheria, Tetanus and Pertussis vaccination:  No coverage: 8.4%; reference  CHW only: 11.6%; 0.7 (0.33;1.67)  FHS: 9.5%; 0.93 (0.45;1.92)  FHS + CHW: 10.0%; 0.86 (0.42;1.75) | No difference in frequency was found in areas covered by the FHS. | Score:41  High quality |
| Lima-Costa, 2013†^49^ | Coverage for flu vaccine in elderly (> 60 years) was the only outcome of interest for our SR | Proportions (p values) | Proportions  Traditional primary Care Unit: 81.8%  Private Health: 82.6%  FHS coverage and use the service: 78.9%  FHS coverage and not use the service: 84.8%  P value NS for all models when compared to traditional primary care unit. | The FHS had no effect in flue vaccination in the different models of care. | Score:35  Low Quality |
| Nery, 2014^50^ | Detection rate of leprosy new cases | Risk ratio (95 % CI) | Risk ratios without BFP  0%-72.02% coverage: 1.00  72.03%-95.06% coverage: 1.02 (0.99;1.05)  >95.06% coverage: 1.09 (1.05; 1.13)  Risk ratios with BFP in the model  0%-72.02% coverage: 1.00  72.03%-95.06% coverage: 1.05 (1.02;1.09)  >95.06% coverage: 1.12 (1.08;1.17) | FHS coverage increased in up to 9% leprosy detection; this effect was leveraged when the BFP was included in the model (12%). | Score:42  High quality |
| Albuquerque, 2007^51^† | Tuberculosis treatment outcomes | Odds Ratio (95 % CI) | Odds Ratio  Treatment Failure:  FHS Yes: 1.00  Other model of primary care: 1.83 (0.84;3.99)  Tuberculosis Death  FHS Yes: 1.00  Other model of primary care: 1.73 (0.87;3.43) | FHS was not associated with better outcomes. | Score:32  Low Quality |
| Benício, 2013^52^† | Child malnutrition | Odds ratio (95 % CI) | Odds ratio  FHS 0-15% coverage: 1.00  FHS 15-30% coverage: 0.62 (0.40;0.96)  FHS 30- 50% coverage: 0.60 (0.38;0.94)  FHS 50- 70% coverage: 0.52 (0.32;0.85)  FHS >70% coverage: 0.55 (0.37;0.81) | In areas with high coverage (>50%) the odds of child malnutrition were around 48% less. | Score:33  Low Quality |

| **Author, year** | **Outcome Studied** | **Measure of effect** | **Main Results** | **Interpretation of main results** | **Quality assessment** |
| --- | --- | --- | --- | --- | --- |
| Araújo, 2012^53^ | Rates of incidence, congenital syphilis | Change in incidence of congenital syphilis with increase unit change in FHS coverage (slope and 95 % CI) | Incidence of Congenital Syphilis per 1000  FHS coverage  Slope: + 0.0035 (0.0003; 0.0067)  Prenatal care coverage  Slope: - 0.0215 (-0.0335; -0.0095) | Congenital syphilis rates were directly correlated with FHS (each 10% increase of FHS coverage was associated whit 0.04/1000 more cases) and inversely correlated with prenatal care. | Score:32  Low Quality |

**Abbreviations**

CHW: Community health works

BFP: *Bolsa família* Program

ICU: Intensive Care Unit

FHS: Family Health Strategy

PCU: Primary care unit

NS: Not significant

CI: Confidence Intervals

**Notes**

†All but references 34, 48, 49, 51, 52 are ecological studies.
